# Tylosin Inhibits *Streptococcus suis* Biofilm Formation by Interacting With the O-acetylserine (thiol)-lyase B CysM

**DOI:** 10.3389/fvets.2021.829899

**Published:** 2022-01-28

**Authors:** Yonghui Zhou, Fei Yu, Mo Chen, Yuefeng Zhang, Qianwei Qu, Yanru Wei, Chunmei Xie, Tong Wu, Yanyan Liu, Zhiyun Zhang, Xueying Chen, Chunliu Dong, Ruixiang Che, Yanhua Li

**Affiliations:** ^1^Heilongjiang Key Laboratory for Animal Disease Control and Pharmaceutical Development, College of Veterinary Medicine, Northeast Agricultural University, Harbin, China; ^2^School of Basic Medicine, Guizhou University of Traditional Chinese Medicine, Guiyang, China; ^3^College of Veterinary Medicine, Heilongjiang Bayi Agricultural University, Daqing, China

**Keywords:** *Streptococcus suis*, biofilms, tylosin, inhibition, O-acetylserine (thiol)-lyase B (CysM)

## Abstract

*Streptococcus suis* (*S. suis*) can decrease its virulence or modify local conditions through biofilm formation, which promotes infection persistence *in vivo*. Biofilm formation is an important cause of chronic drug-resistant *S. suis* infection. The aim of this study was to evaluate whether tylosin effectively inhibits *S. suis* biofilm formation by interacting with O-acetylserine (thiol)-lyase B (CysM), a key enzymatic regulator of cysteine synthesis. Biofilm formation of the mutant (ΔcysM) strain was significantly lower compared to the wild-type ATCC 700794 strain. Tylosin inhibited *cysM* gene expression, decreased extracellular matrix contents, and reduced cysteine, homocysteine, and S-adenosylmethionine levels, indicating its potential value as an effective inhibitor of *S. suis* biofilm formation. Furthermore, using biolayer interferometry technology and fourier-transform infrared spectroscopy, we found that tylosin and CysM could be combined directly. Overall, these results provide evidence that tylosin inhibits *S. suis* biofilm formation by interacting with CysM.

## Introduction

*Streptococcus suis* (*S. suis*) can cause meningitis, septicemia, pneumonia, endocarditis, and arthritis in pigs. Because it is a zoonotic pathogen, it poses a significant harm to public safety (1). In addition, *S. suis* has the ability to form a biofilm that further increases the risk of drug resistance (2, 3). The oral and upper respiratory tracts of pigs, particularly the tonsils and nasal cavities, are important reservoirs of *S. suis* (4). Although most carrier strains are non-virulent, pathogenic strains can colonize the respiratory mucosal surfaces without causing clinical disease, which is the first step in the development of invasive disease in pigs, with subsequent hematogenous and/or lymphogenous dissemination (4).

The intimate contact between *S. suis* and surface structures present on host epithelial cells are critical for resisting innate defense mechanisms and allowing successful competition with resident commensal microorganisms for limited nutritional resources and available space (4). The persistence of *S. suis* in the oral cavity can contribute to increased disease pathogenicity (5). *S. suis* biofilm formation establishes important conditions to ensure its long-term persistence, and decrease its virulence in order to establish long-term infections such as meningitis and endocarditis in its host.

Zhang et al. (6) first demonstrated that *S. suis* biofilm formation contributes to the induction of meningitis using the intracranial subarachnoid route of infection (7) found that *S. suis* decreases its virulence by forming a biofilm which promotes persistence of infection *in vivo*. Biofilms protect bacteria from host and environmental stresses (8). The molecular mechanisms used to perpetuate the virulence of bacteria vary significantly across species, depending on the presence of gene mutations, as well as the nature of proteins that orchestrate biofilm formation (9). All these processes are influenced by the presence of bacterial biofilm-associated proteins (BAP), which drive biofilm formation via their interaction with proteins or enzymes of an already established biosystem. This may completely circumvent the host's entire immune system (10, 11). Extracellular matrix (ECM) formation is also an important factor in regulating the formation of bacterial biofilm, including exopolysaccharides, extracellular DNA, and extracellular protein (12). Following biofilm formation, bacteria present a high degree of drug resistance, and can evade the host's immune response, making infections chronic and difficult to control (13, 14). Therefore, the identification of the regulatory mechanisms involved in *S. suis* biofilm formation as suitable new drug targets have emerged as an important for future research.

*S. suis* biofilm formation is regulated by a variety of factors (15), such as ornithine carbamoyltransferase, autoinducer-2 signaling, and collagen-binding 40 (cbp40) proteins (16). Deletions and overexpression of genes which regulate AI-2 also promote biofilm formation (3). The cysteine biosynthesis pathway, an amino acid metabolism pathway of bacteria, and its associated enzymes, substrates, and products are closely related to biofilm (17). In the cysteine biosynthetic pathway, O-acetylserine (thiol)-lyase B (CysM) is expressed not only under anaerobic conditions, but also catalyzes the synthesis of L-cysteine from O-acetylserine sulfhydrylase (OASS) and thiosulfate (18). Due to the lack of OASS expression in mammals, inhibition of CysM may represent a potential drug target (19). In 1969, researchers successfully isolated and purified the CysM protein from *Salmonella typhimurium*, and analyzed its physical and chemical properties (20). The CysM protein is fold type II pyridoxal 5'-phosphate-dependent enzymes composed of two identical subunits with a total molecular weight of ~68,000 Da. The structure of the CysM isozyme of *Escherichia coli* has been determined and a structural model of its catalytic reaction has been proposed (21). The cysteine produced by bacteria is metabolized to homocysteine by cystathionine-γ-synthetase (metI) and cystathionine-β-lyase (metC). In the methionine cycle, homocysteine, as the substrate of the methionine cycle, generates S-adenosylmethionine under the activity of methionine synthase (metE) and S-adenosylmethionine synthetase (metK). The resulting S-adenosylmethionine is then regenerated by the action of three enzymes, methylase, S-adenosylcysteine ribosidase (mtnN), and S-ribosylhomocysteine lyase (luxS), which are involved in the synthesis of homocysteine, and form AI-2 as a by-product. Thus, we assume that the CysM and cysteine biosynthetic pathways may play important roles in the formation of *S. suis* biofilm (22).

Tylosin is a 16-membered macrolide antibiotic active against both Gram-positive and Gram-negative bacteria (23) which was first obtained from the culture of *Streptomyces fradiae* in 1960 (24). Tylosin is also widely used to prevent *Mycoplasma, Staphylococcus aureus, Pseudomonas aeruginosa*, and *S. suis* infections that cause respiratory disease (25). Macrolides not only exert good antibacterial and growth-promoting effects, but they have also been investigated for their inhibitory activity on biofilm formation (26). Nonetheless, there is little research on the effects of tylosin on *S. suis* biofilm.

Thus, in the present study, we explored the role played by tylosin in the inhibition of biofilm formation of *Streptococcus suis* and the mechanisms involving tylosin inhibitory activity on biofilm formation via the CysM protein.

## Materials and Methods

### Bacterial Strains and Growth Conditions

The *S. suis* wild-type strain ATCC 700794) (27) was exposed to 320 μg/mL tylosin and used to construct the *cysM* (accession number: KX077891.1) deletion strain (ΔcysM) and *cysM* complementary strain (CΔcysM). The specific details for these procedures are provided in the Supplementary File. All strains were grown in Todd-Hewitt broth (THB) or Todd-Hewitt broth agar (THA) supplemented with 5% (v/v) fetal bovine serum, 37°C with agitation.

### Effect of Tylosin on the Growth Rates of *S. suis*

The minimum inhibitory concentration (MIC) of *S. suis* to tylosin was estimated using the microtiter broth dilution method. We previously found that 1/4 MIC of tylosin effectively inhibits *S. suis* biofilm formation (27). Additional experiments confirmed that the wild-type ATCC 700794, mutant (ΔcysM) and complementary (CΔcysM) strains responded similarly to tylosin. In this study, all three strains were treated with and without the 1/4 MIC of tylosin. All strains were incubated at 37°C for 24 h, and the absorbance of the samples was measured every hour at 600 nm (27).

### Crystal Violet Staining and Scanning Electron Microscope

The wild-type ATCC 700794, mutant (ΔcysM) and complementary (CΔcysM) strains were grown in THB medium for 24 h and diluted to 1.0 × 10^6^ CFU/mL in fresh THB. For crystal violet staining, the diluted bacterial solution was diluted 10 times in 100 μL THB medium containing 1/4 MIC, 1/8 MIC, and 1/16 MIC tylosin and inoculated in 96-well tissue culture plates, which were then placed in a 37°C incubator for standing culture for 72 h. A negative control (with THB alone) was also used. Biofilm formation of the mutant (ΔcysM) strain supplemented with 100 and 500 μM cysteine (Sigma Aldrich) were analyzed. The medium, free-floating bacteria, and loosely bound biofilm were removed, and the wells were washed three times with sterile physiological saline. The remaining attached bacteria were fixed with 100 μl of 99% methanol (Guoyao Ltd, China) per well, and after 15 min, plates were emptied and left to dry. Then, plates were stained for 5 min with 100 μl of 2% crystal violet (Guoyao Ltd, China) per well. Excess stain was rinsed off by placing the plate under running tap water. After the plates were airdried, the dye bound to the adherent cells was resolubilized with 100 μl of 33% (v/v) glacial acetic acid (Guoyao Ltd, China) per well. The amount of released stain was quantified by measuring the absorbance at 570 nm with a microplate reader (DG5033A, Huadong Ltd, Nanjing, Jiangsu, China) (28).

For scanning electron microscope (SEM) imaging, the diluted bacterial solution was diluted 10-fold in 2 mL THB liquid medium containing the 1/4 MIC of tylosin was inoculated into 6-well tissue culture plates (containing 0.5 × 0.5 cm sterile frosted glass slide), which were then placed in a 37°C incubator for standing culture for 72 h. A negative control (with THB alone) was also used. SEM micrographs were obtained at the electron microscopy core facility laboratory at the School of Life Sciences, Northeast Agricultural University. The specific procedures for both two experiments have been described (28).

### Determination of Matrix Content in *S. suis* Biofilm by Tylosin

The wild-type ATCC 700794, mutant (ΔcysM) and complementary (CΔcysM) strains were subsequently co-cultured with the biofilm (29). The bacterial concentration was diluted to 1 × 10^6^ CFU/mL, and the diluted bacterial solution was diluted 10-fold in 2 mL THB liquid medium containing the 1/4 MIC of tylosin, and inoculated into 24-well tissue culture plates. These were then placed in a 37°C incubator for standing culture for 72 h. A *S. suis* bacterial solution with the same concentration was inoculated as the control group. After a 72-h incubation, the THB medium was decanted from the 24-well plate, and the biofilm formed at the bottom of the 24-well plate was re-suspended in 3 mL of 0.8% normal saline. The protein was determined by the Bradford method, the DNA was extracted by the Phenol-chloroform method, and the polysaccharide level was determined by the Congo red binding method. The specific experimental steps were performed as described by Desai et al. (29).

### Real-Time Polymerase Chain Reaction

The effects of the 1/4 MIC of tylosin on *cysM, cysE, metI, metE, metK, mtnN*, and *luxS* gene expression were investigated by real-time PCR (RT-PCR). The strains were incubated at 37°C for 24 h. Relative copy numbers and expression ratios of the selected genes were normalized to the expression of the *16S-rRNA* gene (housekeeping gene). The specific primers used in this study are listed in the Supplementary Table. The specific methods used were described in detail in our previous study (27).

### Determination of Cysteine Content

The wild-type ATCC 700794, mutant (ΔcysM) and complementary (CΔcysM) strains were inoculated into 5 mL of THB liquid cultures and incubated under shaking culture at 37°C for 24 h. The bacteria were collected, and the bacterial sample was washed three times with PBS, then centrifuged. The precipitate was re-suspended in 0.5 mL PBS, ultrasonically disrupted, and centrifuged, and the supernatant was collected. A 0.2 mL volume of bacterial supernatant was used to determine cysteine content using a cysteine detection kit (BC0180, Solarbio) according to manufacturer instructions. In summary, 0.3 mL of the extraction solution was added to the bacterial supernatant and was thoroughly mixed, before being centrifuged at 11,000 rpm at 4°C for 10 min. The supernatant was retrieved and reagents 1 and 2 were added to the supernatant, mixed well, and allowed stand for 15 min. The absorbance was then measured at 600 nm, and the process was repeated 3 times. Cysteine reduces phosphotungstic acid to form tungsten blue; thus, an absorption peak was expected at 600 nm. According to the absorbance of cysteine standards with different concentrations at 600 nm, a standard curve was established and was used to determine the cysteine content in the bacterial cultures. Similar cultures of activated bacterial solutions containing the The wild-type ATCC 700794, mutant (ΔcysM) and complementary (CΔcysM) strains in the presence of 1/4 MIC of tylosin were evaluated.

### Determination of Homocysteine and S-Adenosylmethionine Content

The wild-type ATCC 700794, mutant (ΔcysM) and complementary (CΔcysM) strains were inoculated into separate THB liquid cultures. After agitating cultures at 37°C for 24 h, a 10 mL sample of bacteria was collected. The bacterial sample was washed three times with PBS, then centrifuged and the pellet was resuspended in 0.5 mL PBS, ultrasonically disrupted, and centrifuged, and the supernatant was collected. The specific determination methods of homocysteine and S-adenosylmethionine were performed according to the instructions of the homocysteine ELISA kit (YX-080325, Sinobstbio) and the S-adenosylmethionine ELISA kit (YX-190113, Sinobstbio). Samples, standards, HRP-labeled antibodies, chromogenic solution, and stop solution were added to coated wells with homocysteine and S-adenosylmethionine. A colorimetric reaction was positively correlated with homocysteine levels. The absorbance of different concentrations of homocysteine and S-adenosylmethionine was determined at 450 nm to establish a standard curve, which was used to calculate the homocysteine content of the bacterial samples. The activated bacterial solutions of the the wild-type ATCC 700794, mutant (ΔcysM) and complementary (CΔcysM) strains were inoculated into a THB liquid culture medium, in the presence of a tylosin solution with a final concentration of a 1/4 MIC tylosin. The samples were prepared as indicated above and subjected to ultrasonic crushing prior to measuring the homocysteine and S-adenosylmethionine levels.

### Direct Binding Test Between Tylosin and CysM Protein

The *S. suis* CysM protein, with a purity of over 90%, was successfully expressed and purified (Supplementary File). Briefly, a plasmid construct encoding pET30a-cysM was introduced into the BL21 expression strain, and subjected to a shaking culture at 37°C up to the OD600 value of ~0.4–0.6. Next, 1 mM isopropyl-β-D-thiogalactopyranoside (IPTG) was added to induce expression of CysM, and was cultured overnight at 37°C. Meanwhile, a control group without IPTG was also prepared. Control bacteria and induced bacteria were collected, crushed by ultrasonication, and centrifuged, and the whole bacteria solution, supernatant, and precipitate were collected and analyzed by SDS-PAGE. Purification was carried out in two steps, Ni column (GE) purification and molecular sieve chromatography (Superdex75, GE). The three elution steps were performed for the Ni-column (20 mM, 40 mM, and 250 mM imidazole in PBS buffer). All proteins were analyzed by SDS-PAGE at each stage.

CysM concentrations were adjusted to 150 μg/mL after obtaining purified protein, and combined with PBS containing 0.05% Tween-20 and 1 mg/mL BSA, and coupled to the Forte Bio Octet NTA sensor. Tylosin was dissolved in water and diluted twice serially, using a 6-gradient concentration (1,000, 5,000, 25,000, 125,000, 625,000, 3125,000 nM). The tylosin dilution series was added to different wells of the sample plate. The NTA sensor was set to interact with tylosin at different concentrations, and the binding and dissociation curves of tylosin and CysM were determined. The dissociation time was set to 1 min.

### Infrared Spectrum Analysis of CysM Protein in Combination With Tylosin

Fourier-transform infrared spectroscopy (FT-IR) analysis of the protein was performed using the KBr tablet method (30). Pure CysM protein and CysM protein-tylosin lyophilized powders were ground and mixed with KBr powder at a mass ratio of 1:150, and KBr was used to set the scanning background. The scanning parameters were set as follows: scanning range: 4,000–400 cm^−1^, resolution: 4 cm^−1^, and number of scans: 32. OMNIC and Orign 8.5 software were used for deconvolution of the infrared spectrum and multipeak fitting of the infrared spectrum.

### Statistical Analysis

Data were analyzed using the SPSS software program (version 17.0). Continuous numerical data were described as the mean ± standard deviation (SD) and compared between groups using a Student's *t*-test or Wilcoxon test, as appropriate. Statistical significance as set at *P* < 0.05.

## Results

### Effects of Tylosin on the Growth of *S. suis*

**Figure 1:**
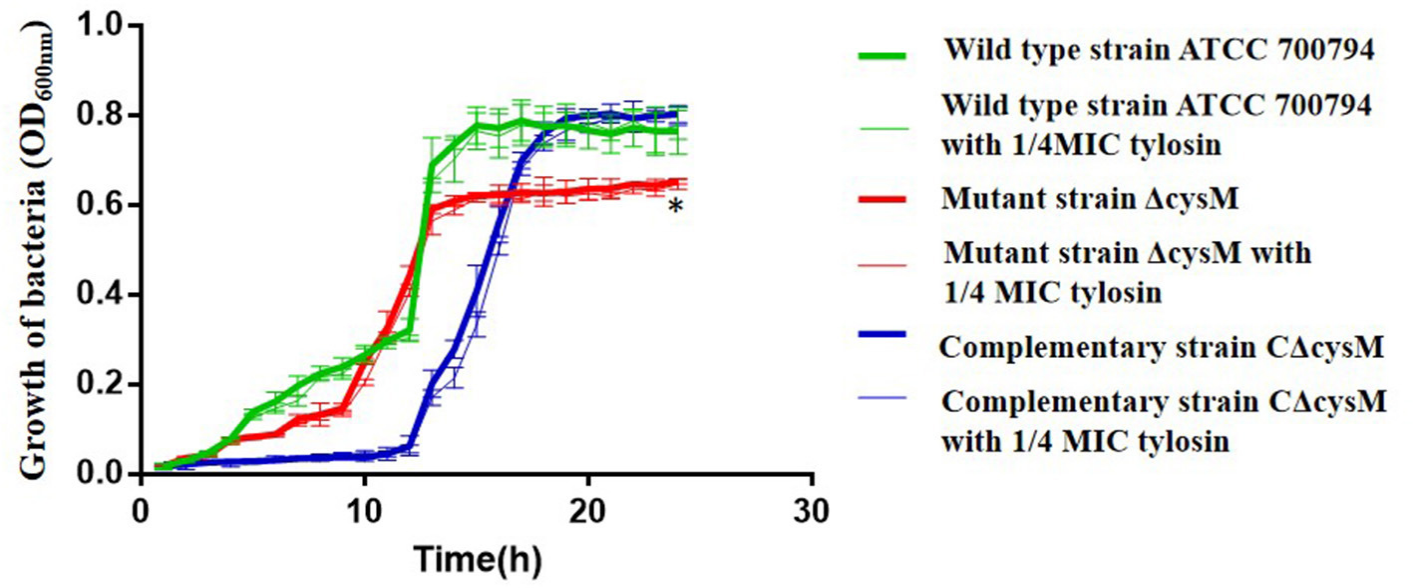
Effects of tylosin on growth curves of *Streptococcus suis*. *P < 0.05 indicates a significant difference between the wild-type strain ATCC 700794 and the mutant (ΔcysM) strain at 24 h.

To evaluate the effects of tylosin on the growth of *S. suis*, we constructed a growth curve using the wild-type ATCC 700794, mutant (ΔcysM) and complementary (CΔcysM) strains (Figure 1). The growth rates of each did not differ in the presence of 1/4 MIC of tylosin. However, the growth rate of the mutant (ΔcysM) strain was lower than that of the wild-type ATCC 700794 strain (*P* < 0.01), and the growth rate of the complementary (CΔcysM) was recovered to that of the wild-type ATCC 700794 strain (*P* > 0.05).

### Inhibition Effect of Tylosin on *S. suis* Biofilm

**Figure 2:**
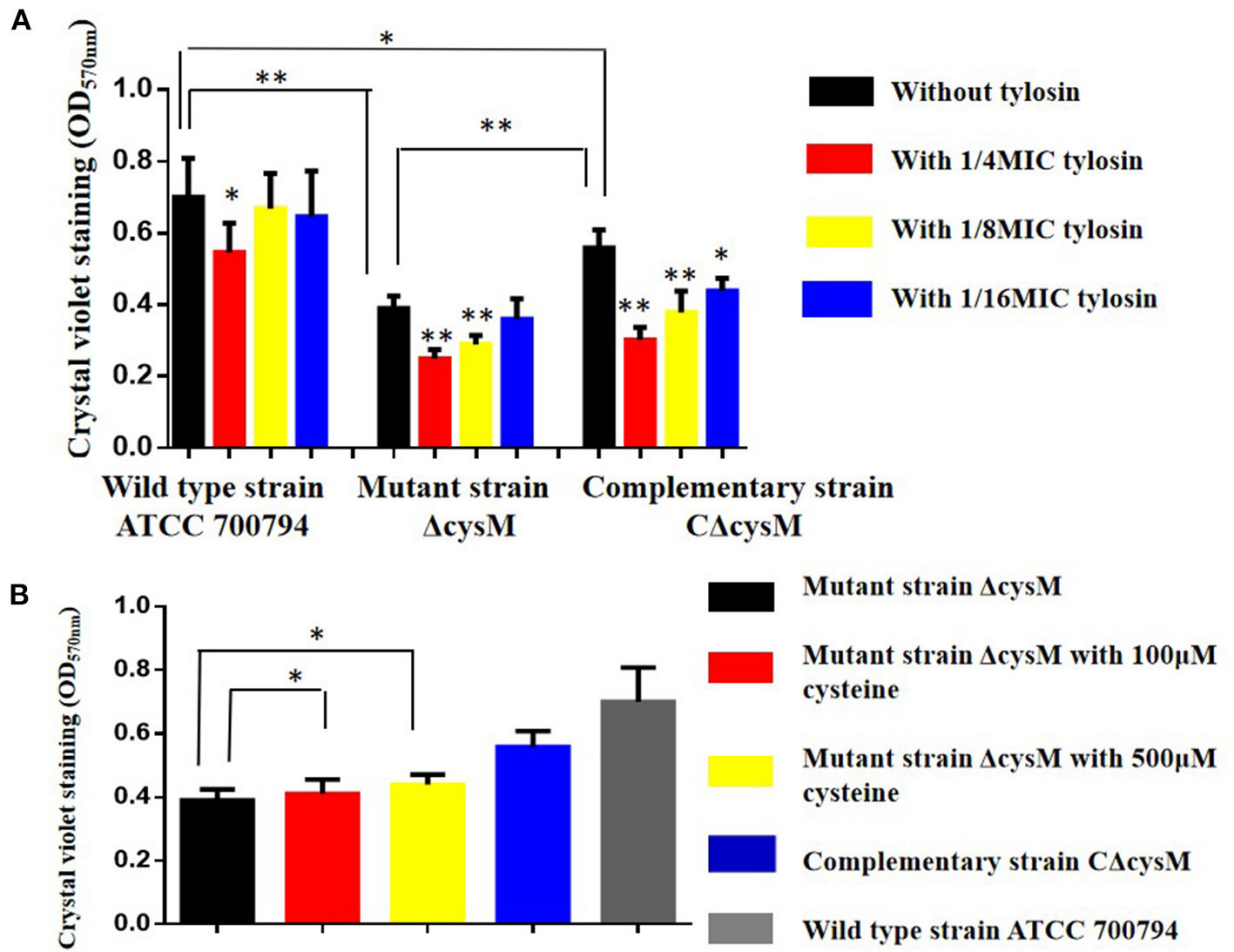
**(A)** Biofilm formation of the wild-type ATCC 700794 strain, the mutant (ΔcysM) strain and the complementary (CΔcysM) strain treated with tylosin or not. **(B)** Biofilm formation of the mutant (ΔcysM) strain treated with cysteine (100 μM and 500 μM) (*P < 0.05 and **P < 0.01 indicate significant difference).

The biofilm formation of the wild-type ATCC 700794, mutant (ΔcysM) and complementary (CΔcysM) strains in the presence of tylosin was determined using crystal violet staining. As shown in Figure 2A, compared to the wild-type ATCC 700794 strain, the biofilm formation associated with the mutant (ΔcysM) strain decreased significantly (*P* < 0.01), while the biofilm formation of the complementary (CΔcysM) strain was restored, although not completely (*P* < 0.05). The biofilm formation of the complementary (CΔcysM) strain was significantly decreased (*P* < 0.05) in the presence of the 1/4 MIC, 1/8 MIC, and 1/16 MIC tylosin concentrations; biofilm formation of the wild-type ATCC 700794 strain was significantly decreased only at 1/4 MIC tylosin (*P* < 0.05). Biofilm formation of the mutant strain ΔcysM was significantly decreased (*P* < 0.01) at both 1/4 MIC and 1/8 MIC tylosin. Furthermore, when the mutant (ΔcysM) strain was treated with varying amounts of cysteine (100 and 500 μM), biofilm formation was restored (*P* < 0.05) (Figure 2B).

### Effects of Tylosin on Biofilm Morphology of *S. suis*

**Figure 3:**
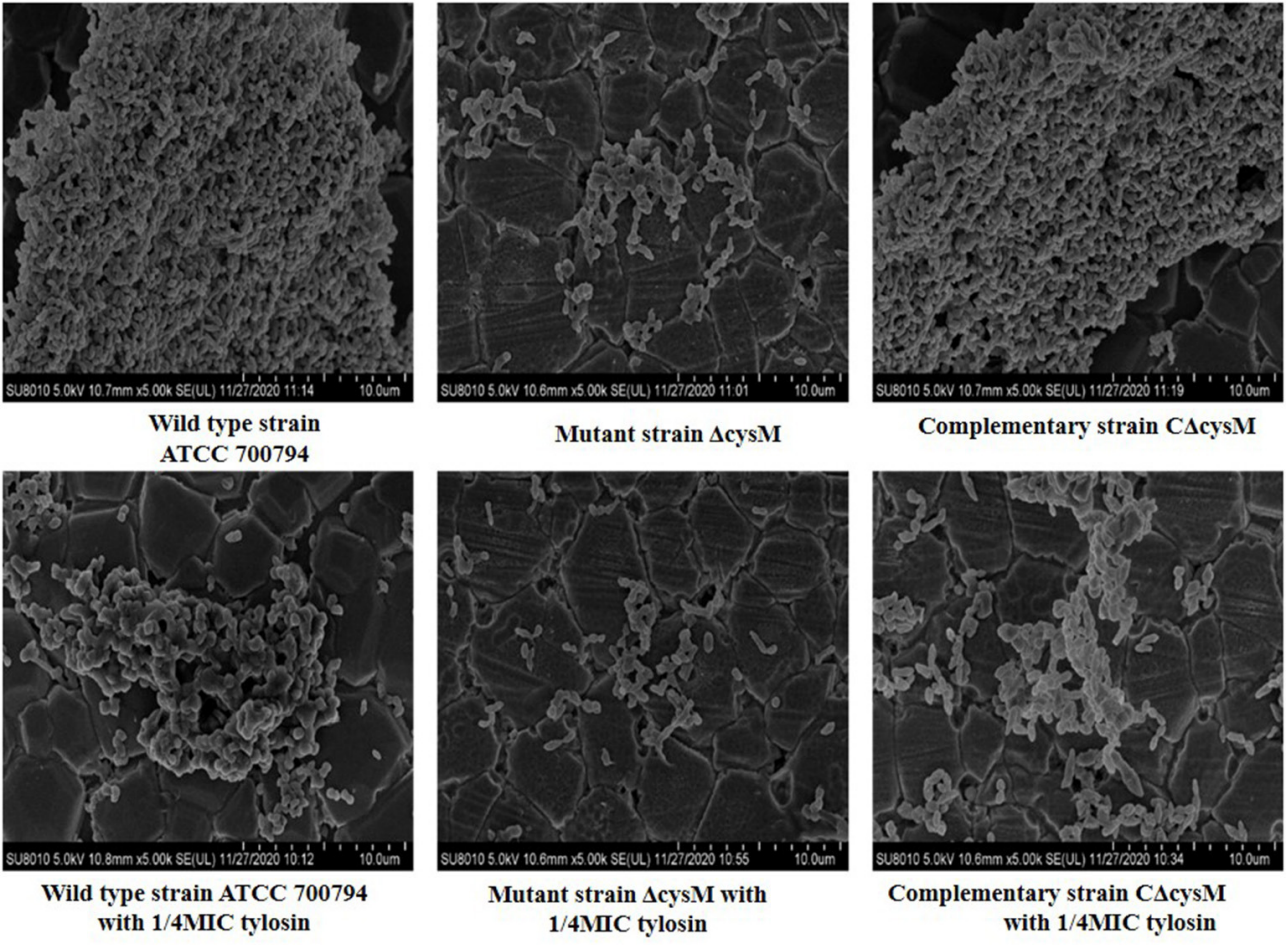
Effect of tylosin on the biofilm morphology of the wild type strain ATCC 700794, the mutant (ΔcysM) strain and the complementary (CΔcysM) strain (Group descriptions are marked below each picture).

Treatment with tylosin at the 1/4 MIC was used to inhibit the biofilm formation of the wild-type ATCC 700794, mutant (ΔcysM) and complementary (CΔcysM) strains and the morphological changes induced in the biofilm morphologies by bacteria were observed by SEM. The wild-type ATCC 700794 strain was closely arranged and adhered to the surface of the cover glass (Figure 3). Its morphology differed from that of the free bacteria and generated a large area of bacterial aggregates to form a mature biofilm. Only a small number of bacteria in the mutant (ΔcysM) strain adhered to the surface of the cover glass; the three-dimensional structure of a mature biofilm could not be formed. The complementary (CΔcysM) strain was closely arranged, and large areas of bacterial aggregates adhered to the surface of the cover glass to form a mature biofilm. However, the overall morphological structure of the biofilm was relatively weaker compared to that of the wild-type ATCC 700794 strain Only a small number of bacterial cells adhered to the surface of the slide, and the morphology of the biofilm of the wild type strain ATCC 700794, the mutant (ΔcysM) strain and the complementary (CΔcysM) strain were inhibited and appeared incomplete in the presence of 1/4 MIC tylosin.

### Effects of Tylosin on the Extracellular Matrix Content in the Biofilm of *S. suis*

**Figure 4:**
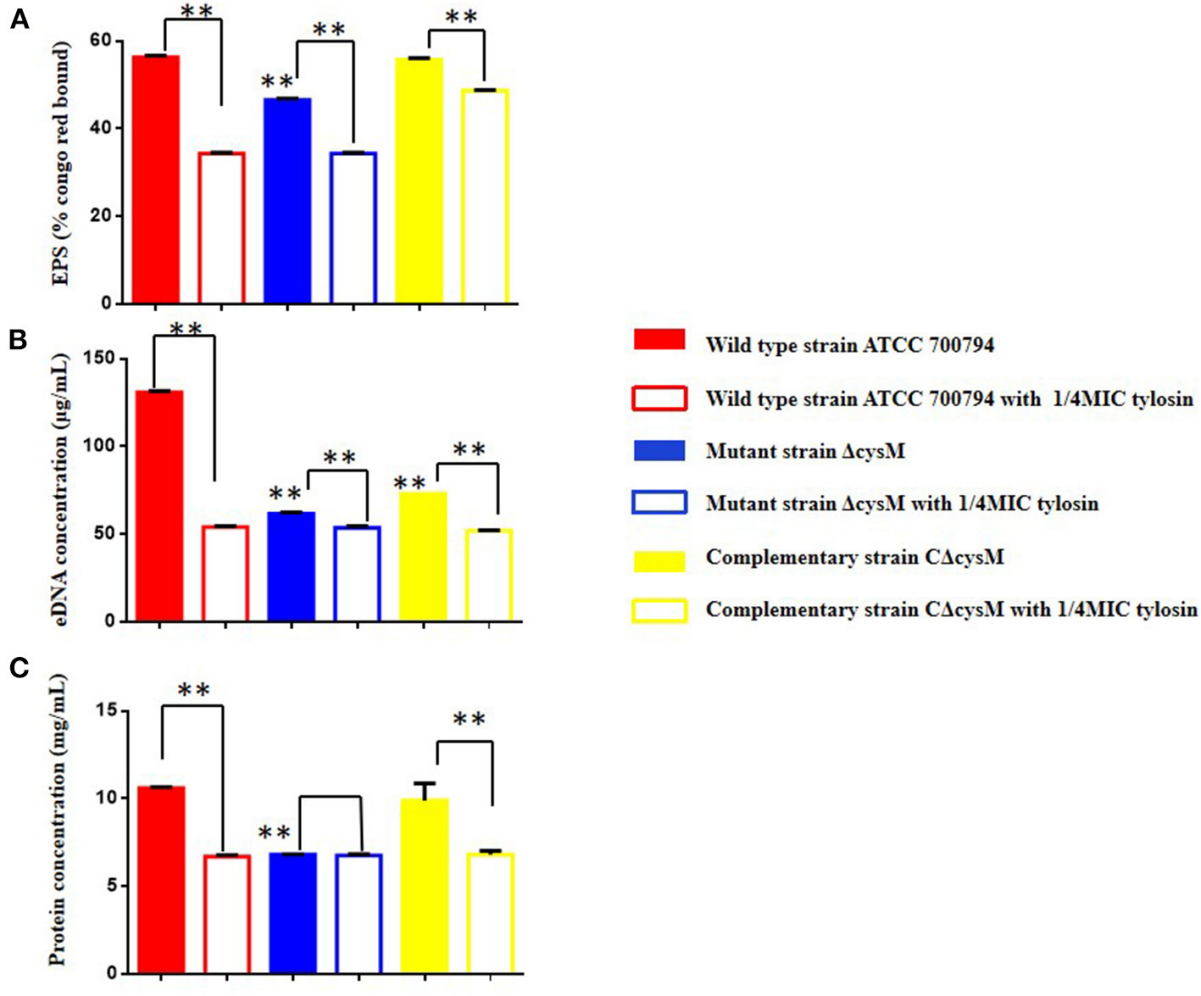
Effect of tylosin on the extracellular matrix of the wild-type ATCC 700794 strain, the mutant (ΔcysM) strain and the complementary (CΔcysM) strain treated or not treated with tylosin. **(A)** Effect of tylosin on the extracellular polysaccharide content; **(B)** effect of tylosin on extracellular DNA content; **(C)** effect of tylosin on extracellular protein content (**P < 0.01 indicate significant difference).

We verified whether tylosin could inhibit biofilm formation by ECM content. As shown in Figure 4A, compared to the wild-type ATCC 700794 strain, the extracellular polysaccharide content of the mutant (ΔcysM) strain biofilm matrix was significantly decreased after *cysM* gene knockout (*P* < 0.01), while the extracellular polysaccharide content was restored in the complementary (CΔcysM) strain (*P* < 0.05); The extracellular polysaccharide contents of the wild-type ATCC 700794 strain, the mutant (ΔcysM) strain and the complementary (CΔcysM) strain were all significantly decreased compared with each respective control group in the presence of 1/4 MIC of tylosin (*P* < 0.01).

As shown in Figure 4B, compared to the wild-type ATCC 700794 strain, the extracellular DNA content in the biofilm matrix of the mutant (ΔcysM) strain decreased significantly after knockout of the *cysM* gene (*P* < 0.01), while the extracellular DNA content was restored in the complementary (CΔcysM) strain (*P* < 0.05), although not completely (*P* < 0.01). Compared to each control group, the extracellular DNA content of the biofilm matrix of the wild-type ATCC 700794, mutant (ΔcysM) and complementary (CΔcysM) strains were all significantly decreased (*P* < 0.01).

As shown in Figure 4C, compared to the wild-type ATCC 700794 strain, the extracellular protein content in the biofilm matrix of the mutant (ΔcysM) strain was significantly decreased (*P* < 0.01), while the extracellular protein content of the biofilm was restored in the complementary (CΔcysM) strain (*P* < 0.05); Compared to each control group, the extracellular protein content in the biofilm matrix of the wild-type ATCC 700794 strain and the complementary (CΔcysM) strain were significantly decreased (*P* < 0.01), while the extracellular protein content in the biofilm matrix of the mutant (ΔcysM) strain was not significantly different (*P* > 0.05).

### Regulatory Effect of Tylosin on Cysteine Metabolism Pathway Genes

**Figure 5:**
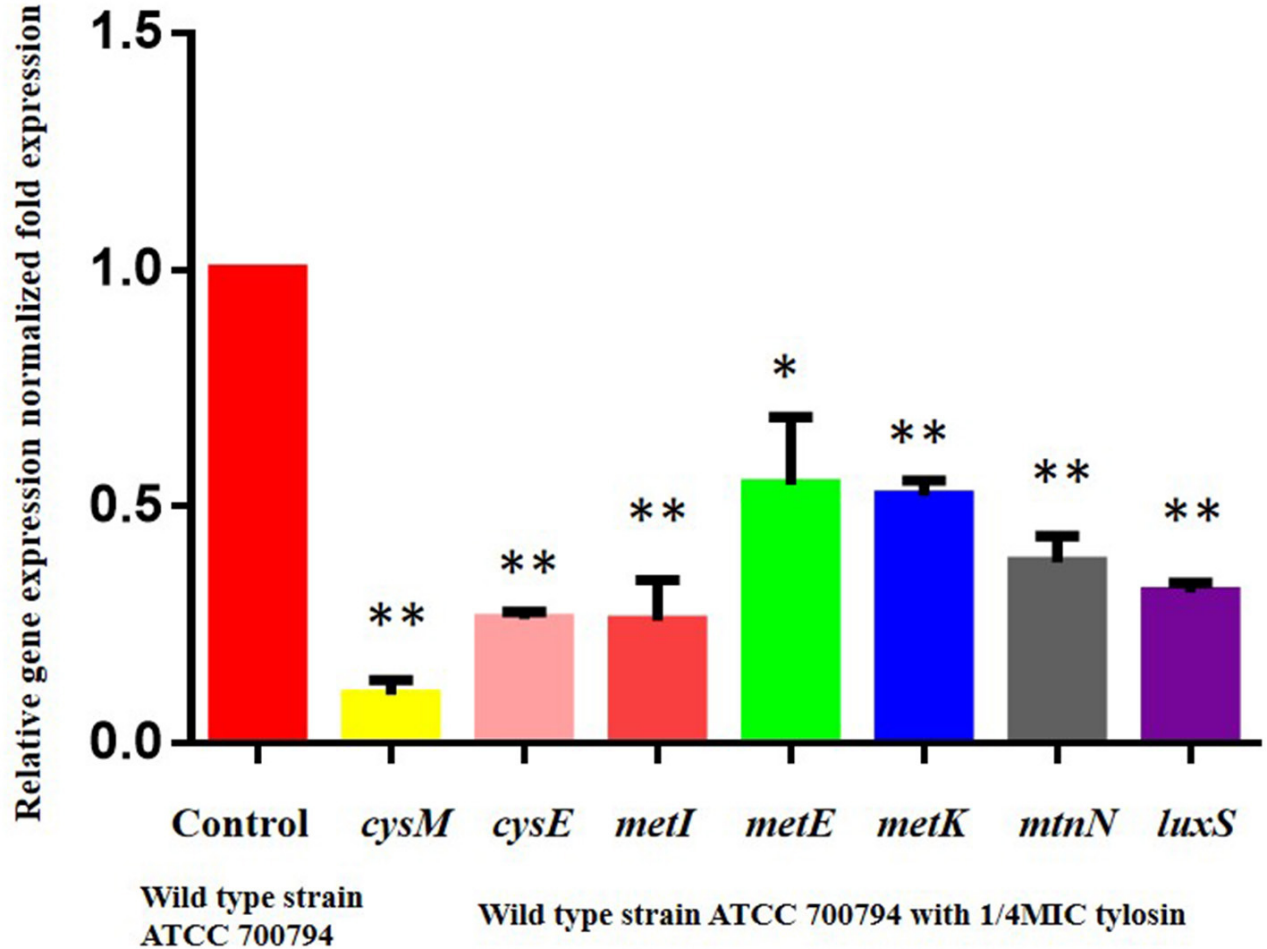
Regulatory effects of tylosin on cysteine metabolism pathway genes. Effect of 1/4 of MIC of tylosin on mRNA expression of the cysteine metabolism pathway genes in the wild-type ATCC 700794 strain (*P < 0.05 and **P < 0.01 indicate significant difference).

We assessed whether tylosin could inhibit biofilm formation by regulating the expression of the cysM, cysE, metI, metE, metK, and mtnN gene. As shown in Figure 5, the expression of the wild-type ATCC 700794 strains cysM, cysE, metI, metE, metK, and mtnN genes was significantly decreased in the presence of 1/4 MIC tylosin (*P* < 0.05).

### Tylosin Regulation of Related Metabolites in the Cysteine Synthesis Pathway

**Figure 6:**
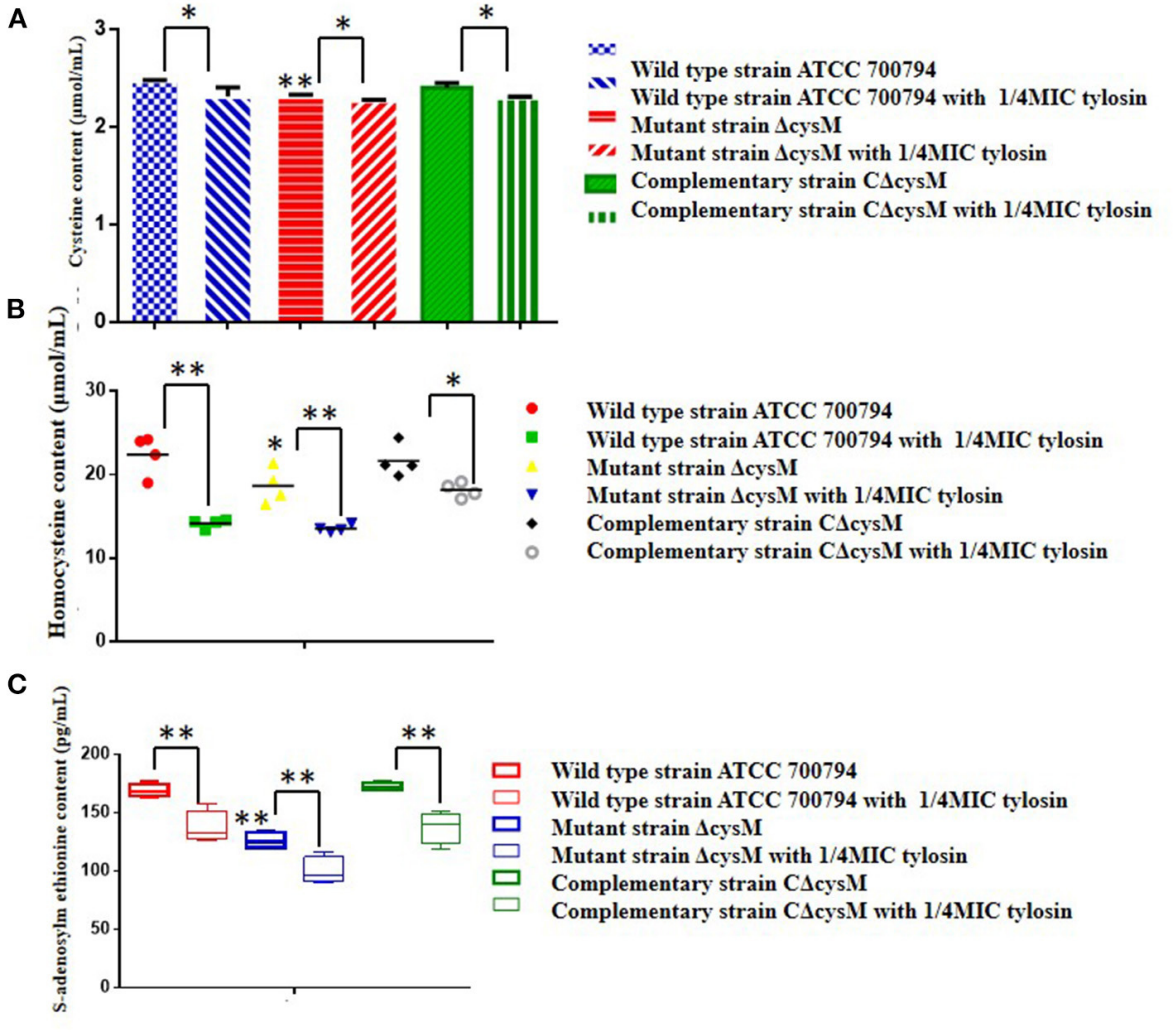
Regulation by tylosin of related metabolites in the cysteine synthesis pathway. **(A)** Effects of tylosin on cysteine content; **(B)** effects of tylosin on homocysteine content; **(C)** effect of tylosin on S-adenosylmethionine content (*P < 0.05 and **P < 0.01 indicate significant difference).

The wild-type ATCC 700794, mutant (ΔcysM) and complementary (CΔcysM) strains were exposed to the 1/4 MIC of tylosin. Compared to the wild-type ATCC 700794 strain, the cysteine content of the mutant (ΔcysM) strain decreased significantly (*P* < 0.01), while the cysteine content was restored in the complementary (CΔcysM) strain (*P* < 0.05) (Figure 6A). Compared to each control group, the cysteine content of the wild-type ATCC 700794, mutant (ΔcysM) and complementary (CΔcysM) strains were markedly decreased in the presence of 1/4 MIC of tylosin (*P* < 0.05).

Compared to the wild-type ATCC 700794 strain, the homocysteine content of the mutant (ΔcysM) strain was significantly decreased (*P* < 0.05), while the cysteine content was restored in the complementary (CΔcysM) strain (*P* < 0.01) (Figure 6B) and compared to each control group, the homocysteine content of the wild-type ATCC 700794, mutant (ΔcysM) and complementary (CΔcysM) strains were all significantly decreased under the activity of the 1/4 MIC of tylosin (*P* < 0.05).

Compared to the wild-type ATCC 700794 strain, the S-adenosylmethionine content of the mutant (ΔcysM) strain significantly decreased (*P* < 0.01), while the cysteine content of the complementary (CΔcysM) strain did not return to levels before knockout of the *cysM* gene (*P* > 0.05) (Figure 6C). The S-adenosylmethionine content of the wild-type ATCC 700794, mutant (ΔcysM) and complementary (CΔcysM) strains were significantly decreased compared to the respective control groups (*P* < 0.01) in the presence of 1/4 MIC of tylosin.

### Expression and Purification of the CysM Protein

**Figure 7:**
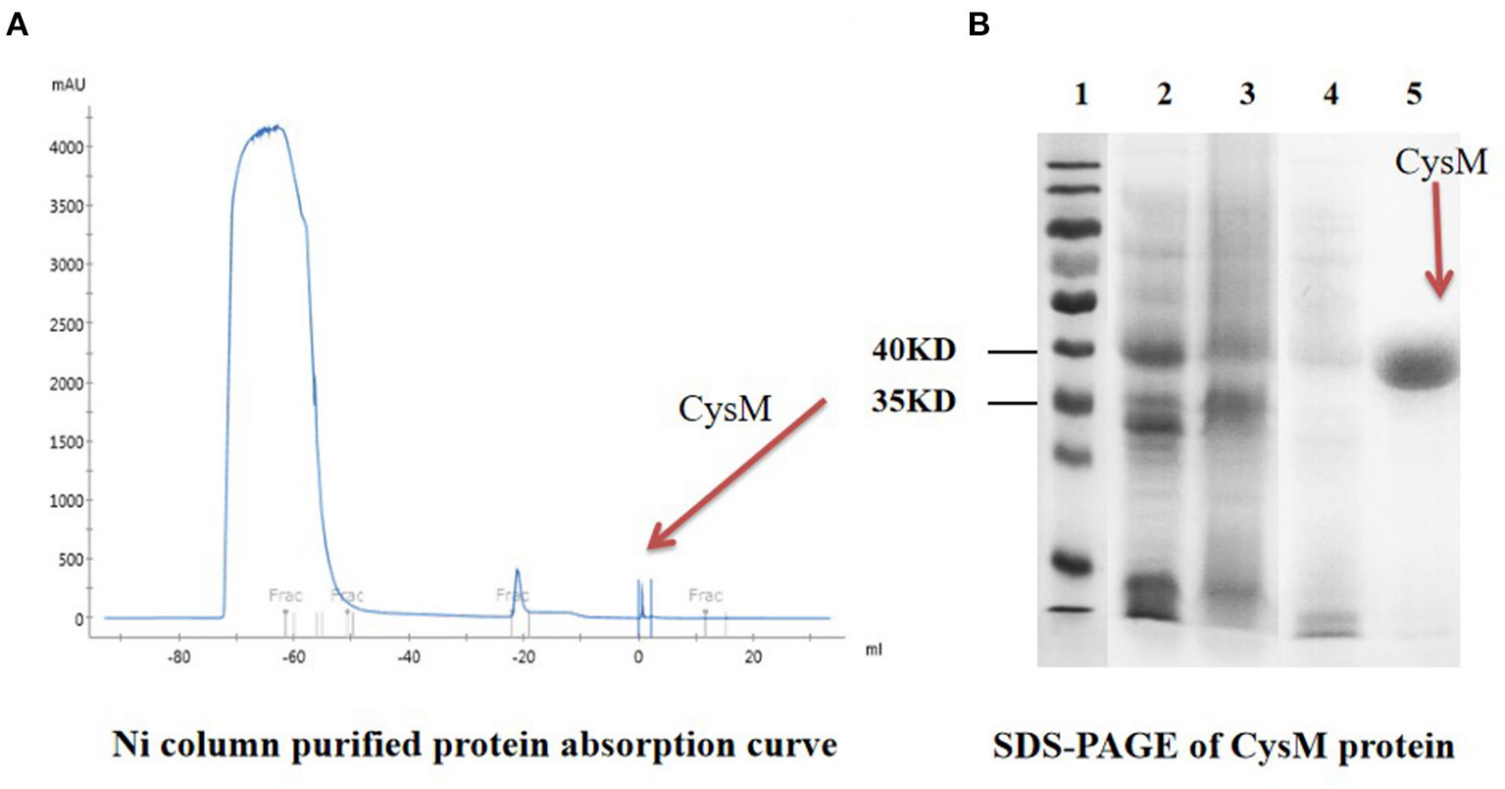
Purification and SDS-PAGE of CysM protein. **(A)** Ni column-purified protein absorption curve; **(B)** SDS-PAGE of CysM protein lane 1: Protein Maker; 2: CysM protein before induction; 3: CysM protein supernatant after induction; 4: CysM protein precipitate after induction; and 5: Purified mature CysM protein.

*S. suis* was induced overnight at 37°C with a 1 mM concentration of IPTG, and the protein produced was expressed and purified by Ni column and molecular sieve chromatography. Following Ni column purification, only one detection peak appeared (The 3rd peak at + 1 mL) (Figure 7), indicating that the protein purity was high. After SDS-PAGE analysis, there was an additional expression band that was consistent with the expected result at about 40 kDa. Purification of the CysM protein was successful and produced a single band, with a purity of >95%. These results indicated that the purified protein met the requirements for subsequent experiments (Figure 7).

### Detection of Direct Interaction Between CysM and Tylosin

**Figure 8:**
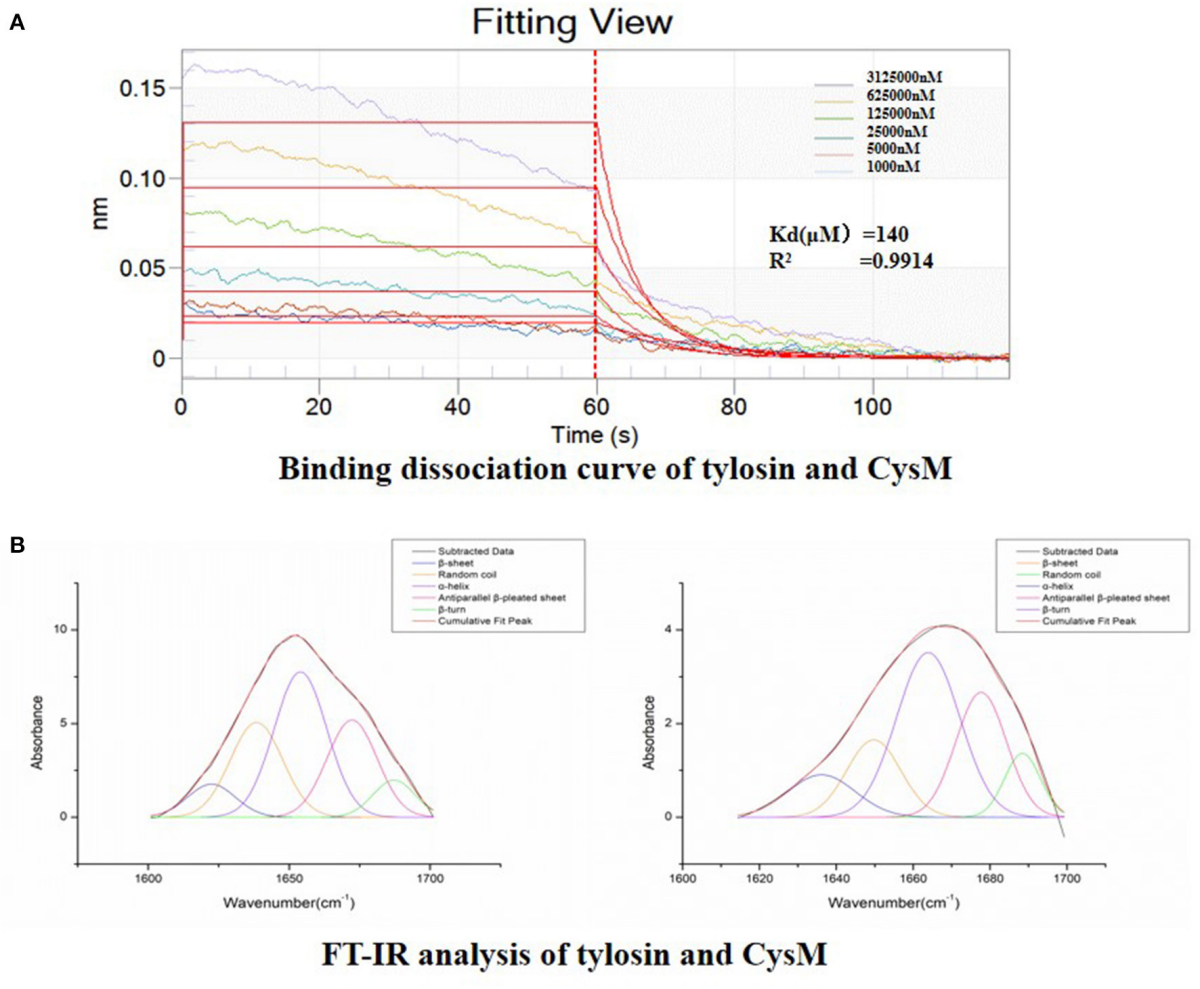
Detection of direct interaction between CysM and tylosin. **(A)** Binding dissociation curve of tylosin and CysM; **(B)** FT-IR analysis of combination between tylosin and CysM.

We verified whether tylosin directly binds to the CysM protein based on a biomacromolecule interaction test and FT-IR analysis. As shown in Figure 8A, the binding of tylosin to the CysM protein reached equilibrium within 60 s, and binding affinity was dose-dependent as the concentration of tylosin increased. The results indicated that tylosin interacted with CysM protein, and that the dissociation equilibrium constant was calculated as Kd = 140 μM. The results indicated that tylosin could directly interact with the CysM protein.

Before and after tylosin acted upon the CysM protein, we obtained the fitting chromatogram of its secondary structure (Figure 8B). Changes in the secondary structure of CysM influenced the changes in the vibration frequency (1,600–1,700 cm^−1^) of the protein amide I band. The structures corresponding to each CysM protein peak included a β-fold, α-helix, β-turn, random coil, and anti-parallel β-fold. To determine the relative percent content of the secondary structure represented by each subpeak, we calculated the relative area of each subpeak. Compared to CysM protein alone, in the absence of tylosin treatment, the composition and content of the secondary structure of CysM protein changed significantly after exposure to the 1/4 MIC of tylosin: the α-helix content increased by 26.38%, the β-sheet content decreased by 8.39%, the β-corner content decreased by 32.66%, the random coil content increased by 15.33%, and the anti-parallel β-sheet content decreased by 0.66%. These results indicated that tylosin interacts with the CysM protein by influencing its secondary structure composition and content and altering its conformation.

## Discussion

The ability of *S. suis* to form a biofilm plays an important role in its virulence and the development of drug resistance (16). This biofilm allows *S. suis* to colonize the respiratory mucosal surfaces without causing overt clinical disease, and facilitates persistence in the oral cavity (4, 5). Furthermore, this biofilm contributes to the induction of meningitis (6). *S. suis* may also decrease its virulence by forming a biofilm able to achieve persistent infection *in vivo* (7). A better understanding of *S. suis* biofilm formation as a pathogenic mechanism and therapeutic target could assist in the prevention and management of *S. suis* infection (16).

In this study, we found that 1/4 of MIC tylosin inhibited *S. suis* biofilm formation; after treatment, the stereo-structures of the biofilm were no longer detectable. Furthermore, 1/4 MIC of tylosin decreased the ECM content (i.e., polysaccharides, DNA and protein) of the *S. suis* biofilm. The mechanisms by which tylosin inhibits the formation of biofilm need to be further explored. However, *S. suis* biofilm formation is known to be regulated by various factors (15).

For example, cysteine biosynthesis pathway plays an important role in the biofilm formation of some bacteria (17). In *Staphylococcus mutans, O-acetylserine sulfatase* (*CysK*) overexpression increases cysteine synthesis, which in turn promotes biofilm formation (31). Furthermore, *in vitro* testing has showed that *CysK* gene deletion inhibits biofilm formation by reducing polysaccharide production (32). Thus, we also explored whether a similar phenomenon occurs in *S. suis*. In this study, we constructed the *cysM* gene deletion mutant (ΔcysM) strain and the *cysM* gene complementary (CΔcysM) strain (Supplementary File). The ability of the mutant (ΔcysM) strain to form a biofilm was reduced, while the ability of the complementary (CΔcysM) strain to form a biofilm was restored. However, compared to the wild-type ATCC 700794 strain, the growth of the mutant (ΔcysM) strain was modified (*P* < 0.01) insofar as CysM no longer influenced *S. suis* biofilm formation.

The latter finding may also be related to other factors, such as the influence of the quorum sensing (QS) system of the mutant (ΔcysM) strain. In previous studies, the QS system was identified as a key factor affecting the formation of *S. suis* biofilm (16). The inability of the complementary (CΔcysM) strain to fully recover the ability to form a biofilm (*P* < 0.05) may also indirectly reflect this phenomenon. However, when the mutant (ΔcysM) strain was treated with varying amounts of cysteine, biofilm formation was restored. Thus, it is not difficult to see that CysM may play an important role in the biofilm formation of *S. suis*.

We also explored whether tylosin inhibits biofilm formation by interfering with CysM and the cysteine biosynthesis pathway, which consists of a sulfuration pathway, an anti-sulfuration pathway, and a methionine cycle. This process generates intermediate metabolites cysteine, homocysteine, and S-adenosylmethionine are very important in the cysteine biosynthesis (33). Tylosin could inhibit *cysM, cysE, metI, metE, metK*, and *mtnN* gene expression and reduce cysteine, homocysteine, and S-adenosylmethionine levels, indicating that tylosin interferes with cysteine synthesis. However, some complex changes appeared in biofilm formation, which were attributed to alterations in the ECM (i.e., polysaccharides, DNA, and protein) and cysteine pathway metabolites (i.e., cysteine, homocysteine and S-adenosylmethionine) after exposure to 1/4 MIC of tylosin in the mutant (ΔcysM) and complementary (CΔcysM) strains. Tylosin thus participated in a complex mechanism to influence *S. suis* biofilm formation, and CysM may not be the only factor.

We also explored the interaction between CysM and tylosin by BLI and FT-IR analysis. BLI is an optical analysis technology that monitors the interaction between biological macromolecules and small ligand molecules in real-time (34). FT-IR is a new method for studying the interaction between drugs and proteins, as well as the relationship between structure and function at the level of secondary protein structural changes (35). The direct interaction between tylosin and CysM might also influence *S. suis* biofilm formation. However, this is only a preliminary exploration of these direct effects. The assessment of protein crystallization, small-molecule activity, and amino acid binding site mutations could provide further insight into the interaction between CysM and tylosin.

In conclusion, this study provided evidence suggesting that tylosin inhibits *S. suis* biofilm formation via its interactions with the CysM protein. However, findings from this study were derived under *in vitro* conditions. The effective serum concentration of tylosin *in vivo* that interacts with CysM may be different compared to *in vitro* conditions. Future studies should perform an the in-depth exploration of CysM and tylosin interactions. It is also necessary to verify the results of the present study *in vivo* using appropriate models.

## Data Availability Statement

The datasets presented in this study can be found in online repositories. The names of the repository/repositories and accession number(s) can be found in the article/Supplementary Material.

## Author Contributions

YL and RC designed the whole study. YZho and FY directed the completion of the experiment. MC, YZha, QQ, YW, CX, TW, YL, ZZ, XC, and CD were supportive during the experiment. All authors contributed to the article and approved the submitted version.

## Funding

This work was supported by National Natural Science Foundation of China (Nos. 31902327 and 32072908).

## Conflict of Interest

The authors declare that the research was conducted in the absence of any commercial or financial relationships that could be construed as a potential conflict of interest.

## Publisher's Note

All claims expressed in this article are solely those of the authors and do not necessarily represent those of their affiliated organizations, or those of the publisher, the editors and the reviewers. Any product that may be evaluated in this article, or claim that may be made by its manufacturer, is not guaranteed or endorsed by the publisher.
