# Supplementary material for: Tylosin Inhibits Streptococcus suis Biofilm Formation by Interacting With the O-acetylserine (thiol)-lyase B CysM
Source: Front Vet Sci. 2022 Jan 28;8:829899. doi: 10.3389/fvets.2021.829899 (PMC8832016; doi:10.3389/fvets.2021.829899)
Supplement: Supplementary file 1 [file Table_1.pdf]

Table Primers for plasmid construction

Oligonucleotide name	Sequence (5' to 3')
*cysM*_A_up	GTCGAC TTATCATGATGAAATGGATGTCAAC
*cysM*_A_down	TTCTCCTTTACTCATATGGCACCTCCAAATAATGT
*cysM*_C_up	GCATGGATGAACTATACAAATAAAAAGAAAGAAGGTTAGGA
*cysM*_C_down	GGATCC TGGGGCATTCGCATCCAGAACTGCA
*cysM*_B _up	ATTTGGAGGTGCCATATGAGTAAAGGAGAAGAACTTTTCACTG
*cysM*_B _down	TAACCTTCTTTCTTTTTATTTGTATAGTTCATCCATG
*cysM*_D_up	CTGCAG ATTTGGAAAGACCTGCTT
*cysM*_D_down	GGATCC TTAATCATTGAACTCATAAAGAGCT
*cysM*_E_up	GGATCC ATGGCTATTTATCAAAACATTACTC
*cysM*_E_down	CTCGAG TTAATCATTGAACTCATAAAGAGCT
cysM-F	ACTGGAAAGGAACCCAGCCA
cysM-R	CGGTCTTGCTTGGGTTGGAG

Note: Primers including the indicated restriction site (underlined)

Experimental method

1. Construction of Δ*cysM* strain

Extraction of genomic DNA The *Streptococcus suis* was inoculated into liquid THB medium, cultured at 37℃ to the late logarithmic phase, and its genomic DNA was extracted. For specific experimental steps reference to TaKaRa bacterial genome extraction kit. The genome was frozen at -20℃.

Upstream and downstream homologous arms of Δ*cysM* strain and PCR amplification of *gfp* gene Using the extracted genomic DNA as template, the upstream and downstream homologous arm fragments of *cysM* gene were amplified by two pairs of upstream and downstream primers of *cysM*_A and *cysM*_C in the table, and the upstream and downstream primers of *cysM*_B were used to amplify gfp gene.

The reaction conditions of upstream and downstream homologous arms of *cysM* gene are as follows:

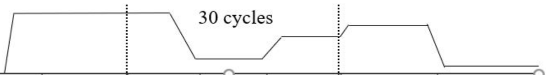

Temp（°C）	98°C	98°C	60°C	68°C	72°C	4°C
Time	2:00	0:10	0:15	1:30	10:00	∞

The reaction conditions of *gfp* fragment of green fluorescent gene are as follows：

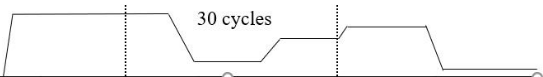

Temp（°C）	98°C	98°C	60°C	68°C	72°C	4°C
Time	2:00	0:10	0:15	1:00	10:00	∞

After amplification, 1 μL of product nucleic acid was taken for electrophoresis detection.

Recovery of PCR products The upstream and downstream homologous arms of *cysM* gene and the amplified products of green fluorescent gene *gfp* were separated by nucleic acid electrophoresis. The target fragment was recovered according to the instructions of TaKaRa DNA gel recovery kit. The concentration and quality of the recovered nucleic acid fragments were analyzed by a nucleic acid analyzer. 1 μL of the recovered fragment was taken for nucleic acid electrophoresis detection.

Construction of cloning vector of Δ*cysM* strain The upstream homologous arm, *gfp* fragment and downstream homologous arm obtained above were ligated by overlap PCR. The primers are shown in the table. The reaction system is as follows:

cysM upstream homologous arm	2 μL
*Gfp*fragment	2 μL
cysM downstream homologous arm	2 μL
Upstream primer	1.5 μL
Downstream primer	1.5 μL
dNTP Mixture	4 μL
5×PrimeSTAR GXL Buffer	10 μL
PrimeSTAR® GXL DNA Polymerase	1 μL

Terilized water	26 μL
Total	50 μL

Overlap PCR reaction conditions are as follows:

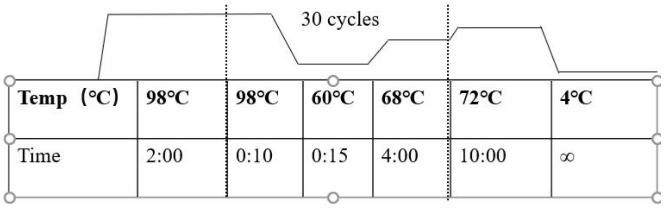

**Temp (°C)**	**98°C**	**98°C**	**60°C**	**68°C**	**72°C**	**4°C**
Time	2:00	0:10	0:15	4:00	10:00	∞

The overlapping PCR amplification target fragment was recovered, and an A tail was added to the 3' end of the gel recovery fragment by using the Mighty TA-cloning Kit to facilitate TA cloning, and then the fragment was cloned into pClone007 Simple Vector cloning vector. The specific reaction system is as follows:

The reaction system with tail A at the 3' end is as follows:

10×A-Tailing Buffer	5 μL
dNTP Mixture	4 μL
A-Tailing Enzyme	0.5 μL
Terminal smooth DNA fragment	10 μL
Sterilized water	30.5 μL
Total	50 μL

Reaction at 72℃ for 20min, standing in ice for 1~2 min. The above-mentioned target fragments were respectively connected with pClone007 Simple Vector cloning vector.

pClone007 Simple Vector	1 μL
A-Tailing DNA fragment	8 μL
10× Topo Mix	1 μL
Total	10μL

Reaction condition：22℃， 5 min.

The ligation product was transformed into competent cells of DH5α *Escherichia coli*

① 10 μL ligation products were all added into 100 μL DH5α competent cells, and the cells were bathed in ice for 30 min.

② After heating at 42℃ for 90 s, put it in ice for 3 min.

③ Add 800 μL LB medium and shake culture at 37℃ for 1 hour.

④ 200 μL of bacterial culture was evenly coated on LB agar plate containing AMP; After standing for 2 hours, culture upside down for 16 hours until a single colony grows, pick out a single colony, and shake culture in an incubator at 37℃.

⑤ The plasmid was extracted by TaKaRa plasmid DNA small purification kit, and the recombinant plasmid pClone007:: *gfp-cysM* was obtained for the next experiment.

Preparation of insert fragment of suicide plasmid *Sal*I enzyme and *BamH*I enzyme were used to carry out double digestion reaction on pSET4s plasmid and pClone007:: *gfp-cysM* plasmid. The reaction system is as follows:

*Sal*I	10 μL
*BamH*I	10 μL
pSET4s/ pClone007:: *gfp-cysM*	20 μL
T×buffer	15 μL
Sterilized water	45 μL
Total	100 μL

After mixing evenly, enzyme digestion was carried out in water bath at 37℃ for 3 hours. The above enzyme digestion products were separated by nucleic acid electrophoresis and the gel was recovered.

Construction of suicide plasmid pSET4s-*cysM* The nucleic acid fragment used to construct the suicide plasmid was ligated with its linear pSET4s suicide plasmid, and the ligation reaction system was as follows:

T4 DNA Ligase	1 μL
10×buffer	1 μL
pSET4s	2 μL
Enzyme-cut fragment	6 μL
Total	10 μL

Mix, and connect overnight in a 16℃ water bath.

The ligation product was transformed into competent cells of DH5α *Escherichia coli* The ligation products were transformed into *E.coli* DH5α competent cells, coated on LB solid medium containing 50 μg/mL Spectinomycin (Spec), and the negative control group was set up, and cultured at 37℃ for 16 hours.

PCR identification of suicide plasmid Single colonies were selected from the transformation plates, and the single colonies of suicide plasmids were inoculated into 5 mL LB liquid medium containing 50 μg/mL Spec, and cultured at 37℃ for logarithmic phase. Then, the DNA of the plasmids was extracted, and the suicide plasmid DNA was used as a template, and two pairs of upstream and downstream primers, *cysM*_B and *cysM*_E, were used for amplification. PCR amplification products were analyzed by nucleic acid electrophoresis.

Suicide plasmid transformed into competent cells of *Streptococcus suis* After the electroporation competent cells of *Streptococcus suis* were prepared, 10 μL of suicide plasmid pSET4s-*cysM* and 100 μL of electroporation competent cells were mixed evenly, and then put into the pre-cooled electroporation cup, ice bath for 30 min, and electric shock. Electrical conditions: 2500 V, 200Ω, 25μF. Immediately after the electric shock, add 5 mL of resuscitation solution and shake culture at 28℃ for 3 h. Finally, 100 μL of shaking culture solution was coated on THB solid medium containing Spec(100 μg/mL), and then cultured at 28℃ for 48 hours, single colony was picked and identified.

Screening of *cysM* gene deletion strains Screening of *Streptococcus suis* gene deletion strains by temperature and pressure. Inoculate the strain into THB medium without spectinomycin, and start subculture. The strain oscillated for 24 hours at 28℃, then transferred to the culture medium, and then oscillated for 24 hours at 37℃. This process is the first generation screening. Bacteria are subcultured every two days. The bacteria in the 7th,

9th and 11th rounds were screened by flow cytometry to determine whether the *cysM* gene deletion strain was successfully construction. Specific steps are as follows:

(1) The above-mentioned identified screening strains and negative control strains are subjected to resuscitation culture, that is, shaking culture at 37℃.

(2) Under aseptic condition, take 1 mL of bacteria solution and centrifuge at 12000 r/min for 2 min.

(3) Discard the supernatant, suspend the bacteria with 1 mL sterile PBS(pH=7.4) for 12000 r/min for 2 min, and repeat twice.

(4) Finally, 500 μL sterile PBS(pH=7.4) was used to suspend bacteria for sorting bacteria by flow cytometry.

(5) After adjusting the wavelength of flow cytometer to 488 nm, detect it. Compared with the negative control group, the last 1% cells with the highest fluorescence intensity are sorted out, placed in sterile 96-well plate containing THB medium, and cultured at 37℃ for 24-48 hours.

PCR identification of Δ*cysM* strain The above-mentioned sorted monoclonal strains were identified by PCR, and the genomic DNA of the sorted strains with *cysM* gene deletion was used as template to amplify them by PCR with *cysM*_E primer and *cysM*_B primer, and the PCR products were tested by agarose gel electrophoresis.

2. Construction of CΔ*cysM* strain

Using genomic DNA as template, the upstream and downstream primers of *cysM*_D in the table were used to amplify the gene complement fragment of *cysM*.

Construction cloning vector of CΔ*cysM* strain The gel was recovered, and the tail A was added to the 3' end with Mighty TA-cloning Kit, and the ligated product was cloned into pMD19-T-simple cloning vector. The specific system is as follows:

pMD19-T-simple	1 μL
A-Tailing DNA	4 μL
Solution I	5 μL
Total	10 μL

After reacting at 16°C for 4 hours, the ligation product was transformed into competent cells of DH5α *Escherichia coli.*

Preparation of insertion fragment of complement plasmid

*Pst*I and *BamH*I were used to digest the pSET2 plasmid and the correctly identified pMD19T-CΔ*cysM* plasmid. The reaction system was as follows:

*Pst*I	10 μL
*Bam*HI	10 μL
pSET2/pMD19T-CΔ*cysM*	20 μL
K×buffer	10 μL
Sterilized water	50 μL
Total	100 μL

After mixing evenly, enzyme cut at 37℃ for 3 hours. The above enzyme digestion products were separated by nucleic acid electrophoresis and the gel was recovered.

Construction of complementary plasmid pSET2-CΔ*cysM* The gel recovery fragment used to construct the patch plasmid was ligated with the linear pSET2 vector, and the reaction system was as follows:

T4 DNA Ligase	1 μL
10×Buffer	1 μL
pSET2	3 μL
Enzyme-cut fragment	5 μL
Total	10 μL

After mixing, connect overnight in water bath at 16℃ and transform to DH5α competence.

Construction and Screening of CΔ*cysM* strain The patch plasmid was transferred into the competent cells of Δ*cysM* strain, and the patch strain containing pSET2 plasmid was finally obtained through spectinomycin resistance screening. Inoculate the strain into liquid THB culture medium for amplification, culture at 37℃ to the late logarithmic phase, extract DNA template, and freeze at -20℃ for later use.

PCR identification of CΔ*cysM* strain Using the above DNA as template, the *cysM* gene complementary strain obtained in the above steps was identified by PCR using the

upstream and downstream primers of *cysM*_D and *cysM*_E in the table, and the PCR products were tested by agarose gel electrophoresis.

3. Expression of CysM protein

PCR amplification of *cysM* fragment The extracted genomic DNA was amplified by PCR using the primers *cysM* _ E_ up and *cysM*_E_down expressed by CysM protein in the table. The amplification system and amplification conditions refer to the above experimental steps. The reaction products of PCR were separated by nucleic acid electrophoresis and recovered by gel.

Construction of recombinant plasmid pMD19T-*cysM* An A tail was added at the 3' end of the gel recovery product and the *cysM* fragment was ligated to the clone vector. Then, the above products were converted into competent cells of DH5α, and the positive recombinant plasmid cloned into pMD19-T-simple vector was identified, finally obtaining the pMD19T-*cysM* recombinant plasmid.

Construction of expression plasmid The constructed pMD19T-*cysM* recombinant plasmid and the prokaryotic expression vector plasmid of pET30a were subjected to double enzyme digestion reaction by *BamH*I and *Xho*I, and the target fragment linearized after enzyme digestion was connected with the vector by T4 DNA Ligase. Then, the ligation product was introduced into DH5α competent cells to amplify the plasmid, and finally pET30a-*cysM* was obtained.

Induced expression and purification of expression plasmid The constructed pET30a-*cysM* plasmid was introduced into BL21 expression strain. After shaking culture at 37℃ until the OD600 value was 0.4~0.6, 0.25 mM and 1 mM isopropyl -β-D-thiogalactopyranoside (IPTG) were added to it, and the expression was induced at 37℃ for 4 hours. Another control group without IPTG was set up. The control bacteria and inducing bacteria were collected, crushed by ultrasonic and centrifuged, and the whole bacteria solution, supernatant and precipitate were collected and analyzed by SDS-PAGE. The CysM protein was purified by Ni column (GE) purification and molecular sieve chromatography (Superdex75, GE), and the protein after each stage was analyzed by SDS-PAGE.

Results

PCR amplification of upstream and downstream homologous arms of *cysM* gene

The upstream homologous arm of cysM gene is 1211bp, and the downstream homologous arm of *cysM* gene is 1099 bp, which is consistent with the theoretical value, as shown in Figure 1:

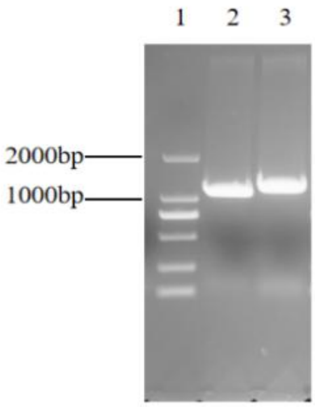

Note：1：DL2000 DNA maker；2：Downstream homologous arm of *cysM* gene；3：Upstream homologous arm of *cysM* gene

Figure 1 PCR amplification of upstream fragment and downstream fragment of *cysM* gene

PCR amplification of *gfp* gene The 717 bp gene fragment was amplified from pMD18-T Vector containing gfp, and the result is shown in Figure 2.

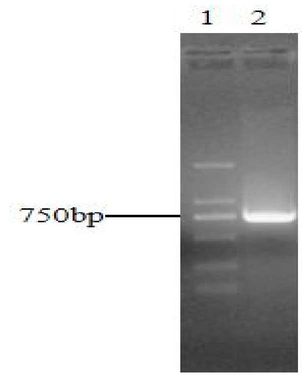

Note：1：DL 2000 DNA maker；2：*gfp* gene

Figure 2 PCR amplification of *gfp* gene

Identification of recombinant cloning plasmid pClone007:: *gfp-cysM* The upstream and downstream homology arm genes and *gfp* genes were ligated by overlap PCR and cloned into the pClone007 Simple vector, and then PCR was performed to identify them. As shown in Figure 3, there is an entry band at 3027 bp, which is as expected. The nucleotide bands match the size. At the same time, after digestion with *Sal*I and *BamH*I, a pClone007 Simple vector fragment of about 1869 bp and a target gene fragment of about 3027 bp were obtained, which were consistent with the expected results. The recombinant clone plasmid pClone007:: *gfp-cysM* was successfully constructed.

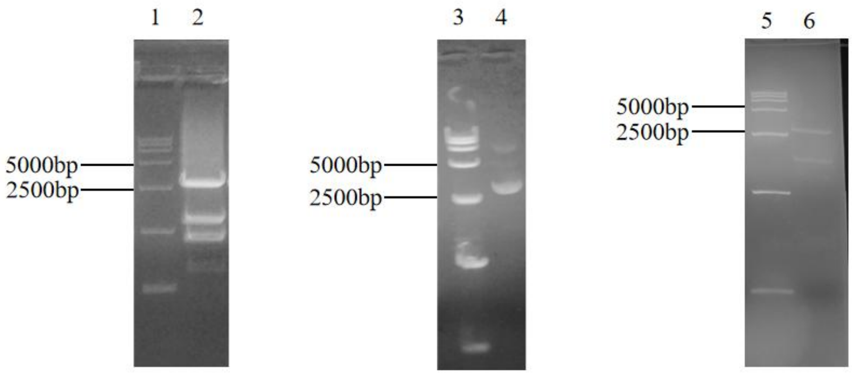

Note: 1: DL15, 000DNA marker; 2: Upstream homology arm-*gfp*-downstream homology arm overlap PCR results; 3: DL15, 000DNA marker; 4: Recombinant plasmid pClone007:: *gfp-cysM* PCR identification results; 5: DL15, 000DNA marker; 6: Recombinant plasmid pClone007:: *gfp-cysM* restriction digestion results

Figure 3 Identification of pClone007:: *gfp-cysM*

Construction of suicide plasmid pSET4s-*cysM* The positive recombinant plasmid pClone007:: *gfp-cysM* plasmid and pSET4s vector plasmid were digested with *Sal*I and *BamH*I, and the target digested fragment was ligated with the digested fragment of pSET4s plasmid, and the obtained recombinant plasmid was identified by PCR. The result showed the target band and insert. The lengths of the target fragments are consistent, as shown in Figure 4, confirming the successful construction of the positive recombinant plasmid suicide plasmid pSET4s-*cysM*.

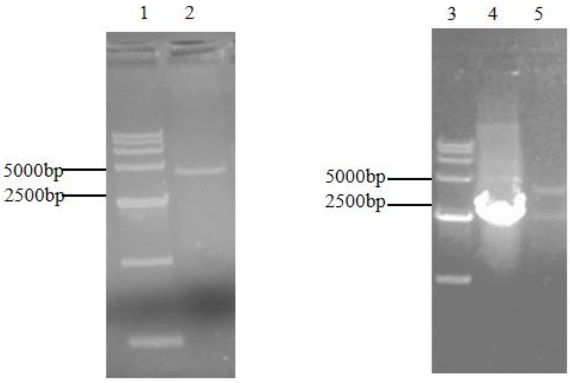

Note: 1: DL15, 000DNA marker; 2: pSET4s digestion result; 3: DL15, 000DNA marker; 4: Recombinant plasmid pSET4s-*cysM* PCR identification results; 5: Recombinant plasmid pSET4s-*cysM* double restriction digestion results

Figure 4 Identification of pSET4s-*cysM*

Screening of Δ*cysM* strain The pSET4s-*cysM* positive plasmid was introduced into the competent *Streptococcus sui*s. Under temperature and pressure, the suicide plasmid and the bacterial genome had homologous recombination, and the *cysM* gene was replaced by the *gfp* gene. As shown in Figure 5, under the excitation wavelength of 488 nm, the fluorescence signals of the seventh, ninth and eleventh generation strains containing GFP tags were detected by flow cytometry. Increase, the GFP fluorescence signal gradually increases. Among them, the fluorescence intensity of the 7th generation is 0.9%, the fluorescence intensity of the 9th generation is 3.1%, and the fluorescence intensity of the 11th generation is 12.8%. In the 11th generation, a strong fluorescence signal is obtained, as shown in Figure 6. It can be used for monoclonal sorting test.

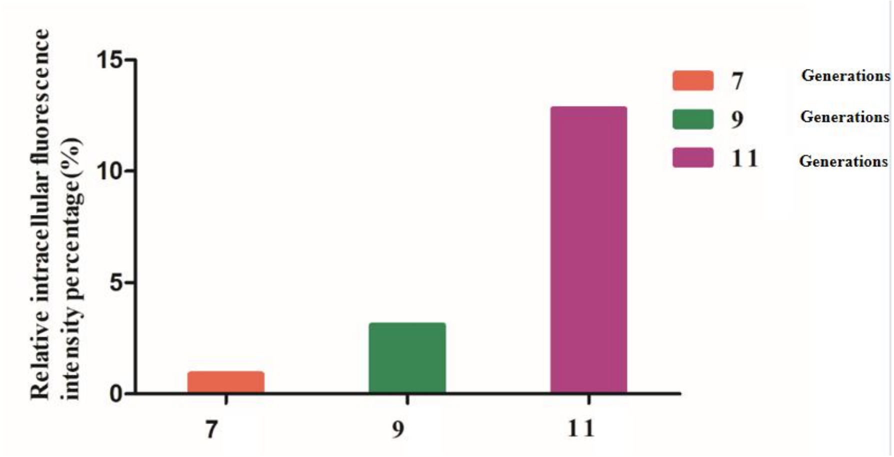

Figure 5 Fluorescence intensity of *S. suis* after temperature-sensitive

screening in different generations

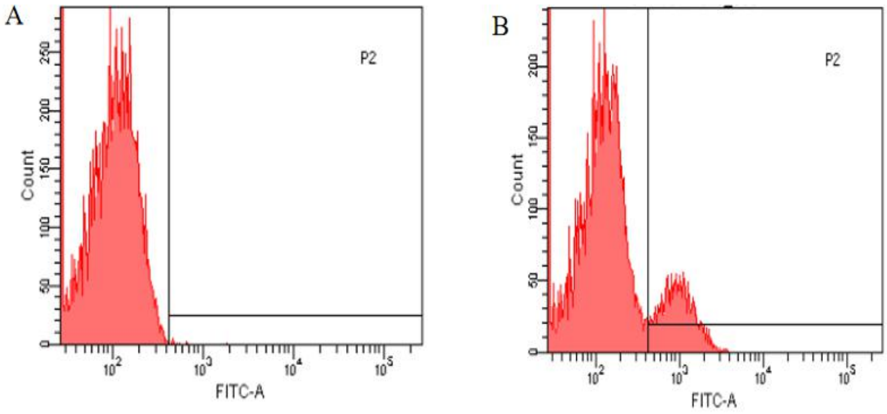

Note：A：Negative control；B：FCM Screening of the 11th Generation Strain

Figure 6 Screening *cysM* gene deletion strain by FCM

PCR identification of Δ*cysM* strain The eleventh generation gene-deleted strains were subjected to a monoclonal sorting test using flow cytometry. After 252 monoclonal strains were obtained, they were identified by two pairs of primers, *cysM*_B and *cysM*_E. As shown in Figure 7, the gene-deleted strain did not amplify the *cysM* gene band; at the same time, the gene-deleted strain amplified the target band of the *gfp* gene, indicating that the *cysM* gene-deleted strain has been successfully constructed.

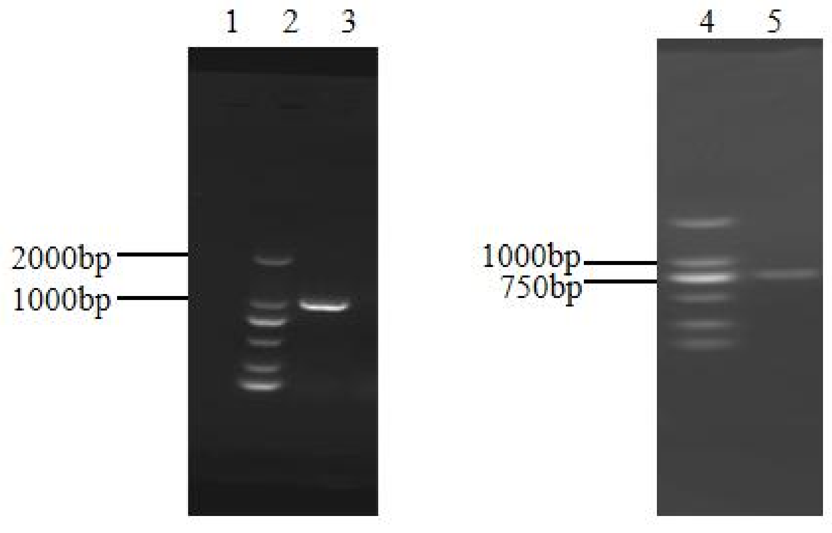

Note: 1: *cysM* gene PCR identification results of *cysM* gene deletion strain; 2: DL2000 DNA marker; 3: Positive control *cysM* gene PCR results; 4: DL2000 DNA marker; 5: *cysM* gene deletion strain *gfp* gene PCR identification results

Figure 7 PCR identification results of *S. suis cysM* gene deletion strain

Construction and identification of CΔ*cysM* strain

Construction of CΔ*cysM* strain The 1401 bp gene fragment of *cysM* containing the promoter was cloned into plasmid pMD19-T-simple, and it was connected to pSET2 by double digestion with *pst*I and *BamH*I, and the constructed plasmid was identified by PCR, as shown in Figure 8, indicating that it is back The complement plasmid pSET2-CΔ*cysM* was successfully constructed.

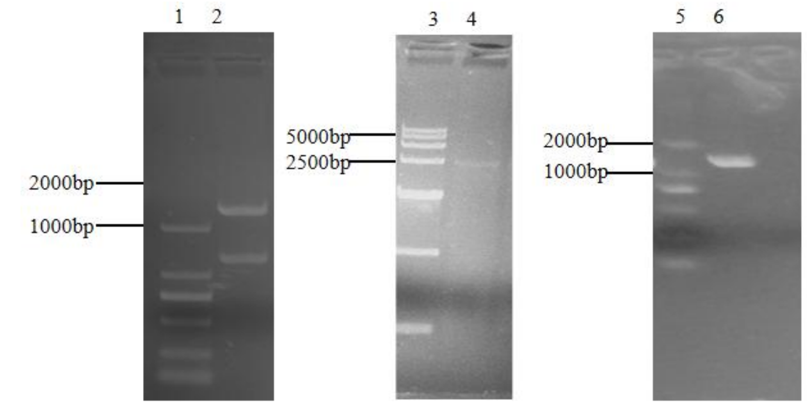

Note：1：DL2000 DNA marker；2：pMD19T-CΔ*cysM* digestion result；3：DL15，000 DNA marker；

4：pSET2 digestion result；5：DL2000 DNA marker；6：pSET2-CΔ*cysM* PCR identification results

Figure 8 Identification results of *S. suis cysM* gene complementary strain

Identification of CΔ*cysM* strain The competent cells of the *cysM* gene deletion strain successfully introduced into the pSET2-CΔ*cysM* plasmid were spread on a THB plate containing spectinomycin resistance, and a single colony was picked and cultured in 5 mL of resistant liquid medium. The next day, two pairs of primers, *cysM*_E and *cysM*_D, were used for PCR identification, as shown in Figure 9, to obtain the target fragments of the *cysM* gene and the complementing gene, respectively. The obtained positive strain is the CΔ*cysM* strain.

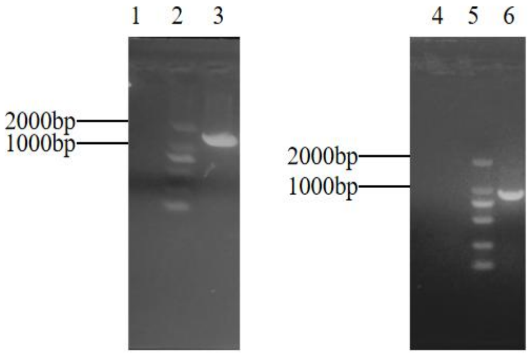

Note：1：PCR identification results of the filling gene of the *cysM* gene deletion strain；2：DL2000 DNA marker；3：PCR identification results of the complementary fragments of *cysM* gene；4：*cysM* gene PCR identification results of *cysM* gene deletion strain；5：DL2000 DNA marker；6：cysM gene PCR identification results of CΔ*cysM* strain

Figure 9 PCR identification results of *S. suis cysM* gene complementary strain

Expression and purification of CysM protein

Molecular cloning of *cysM* gene Using the extracted *Streptococcus suis* genome as a template, the PCR amplification reaction was carried out using the *cysM*_E upstream and downstream primers. The PCR product obtained is as shown in (Figure 10). The band position is about 927 bp, and the preliminary identification is correct. The pMD19-T-simple vector was introduced into the cloning bacteria, and the recombinant cloning plasmid pMD19T-*cysM* of *E. coli* was successfully constructed.

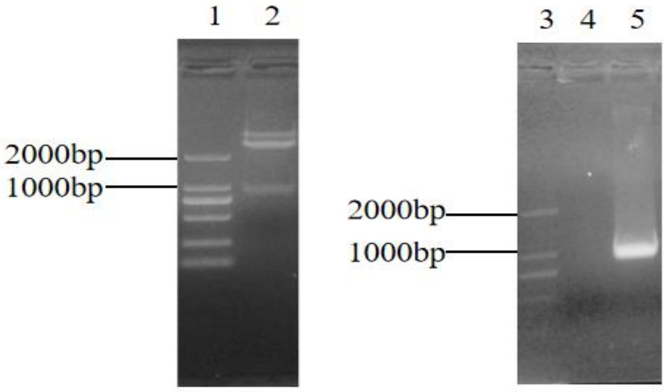

Note： 1： DL2000 DNA marker； 2： Restriction digestion results of pMD19T-*cysM* plasmid； 3： DL2000 DNA marker； 4： PCR negative control of pMD19T-*cysM*； 5： PCR results of the recombinant clone plasmid pMD19T-*cysM*

Figure 10 Construction of pMD19T-*cysM* expression vector

Construction of pET30a-*cysM* expression vector The positive recombinant plasmid pMD19T-*cysM* was double digested with *BamH*I and *Xho*I, and the *cysM* fragment of about 927bp was recovered with a DNA gel recovery kit. Introduced into *Escherichia col*i DH5α by pET30a, and used the extracted plasmid as a template for PCR identification. The obtained target band was consistent with the expected inserted target fragment length. That is, the recombinant clone plasmid pET30a-*cysM* of Escherichia coli was successfully constructed and amplified. The result is shown in Figure 11. It was confirmed that the positive recombinant plasmid pET30a-*cysM* was successfully constructed. Subsequently, the *E. coli* BL21 recombinant strain introduced into the pET30a-*cysM* recombinant plasmid was identified by PCR. There was an entry band at about 927 bp, which was in line with expectations, confirming that the pET30a-*cysM* recombinant plasmid had been successfully transferred into *E. coli* BL21.

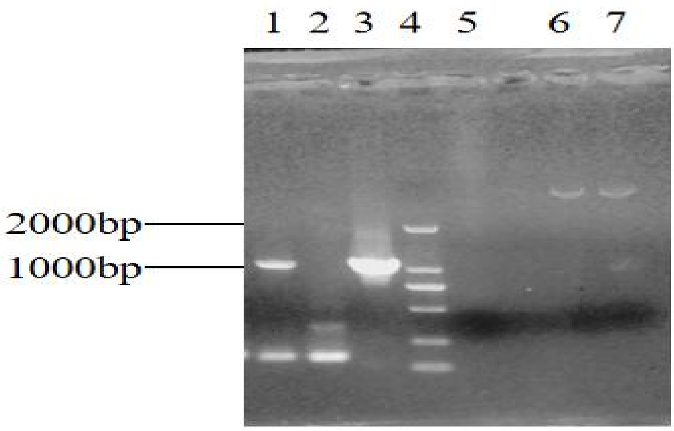

Note: 1: PCR identification of BL21 bacterial liquid containing pET30a-*cysM* plasmid; 2: Negative control (BL21 bacterial solution without pET30a-*cysM* plasmid) PCR results; 3: PCR result of recombinant plasmid pET30a-*cysM*; 4: DL2000 DNA marker; 5: Negative control (PCR result of recombinant plasmid pET30a-*cysM*); 6: Double enzyme digestion results of pET30a vector plasmid; 7:Double restriction digestion results of recombinant plasmid pET30a-*cysM*

Figure 11 Construction of pET30a -*cysM* expression vector

Expression and purification of CysM protein The positive bacteria were induced for 4 h at 37°C and the concentration of IPTG was 0.25 mM and 1 mM respectively. The culture was aspirated and prepared into SDS-PAGE electrophoresis samples, and analyzed by SDS-PAGE with 15% separation gel. It was found that there was a new expression band in line with the expected result at about 40 kD, which confirmed the expression of pET30a-*cysM* protein. After sonication and centrifugation, the precipitate and supernatant were taken for electrophoresis. The results showed that the target protein was expressed in the supernatant, and the protein expression was higher under the conditions of 1 mM IPTG induction (Figure 12). The supernatant of 1 mM IPTG-induced bacteria was purified with AKTA. After purification, a CysM mature protein with good band uniformity and a purity of more than 95% was obtained. These results indicate that our purified protein meets the requirements of subsequent experiments (Figure 13).

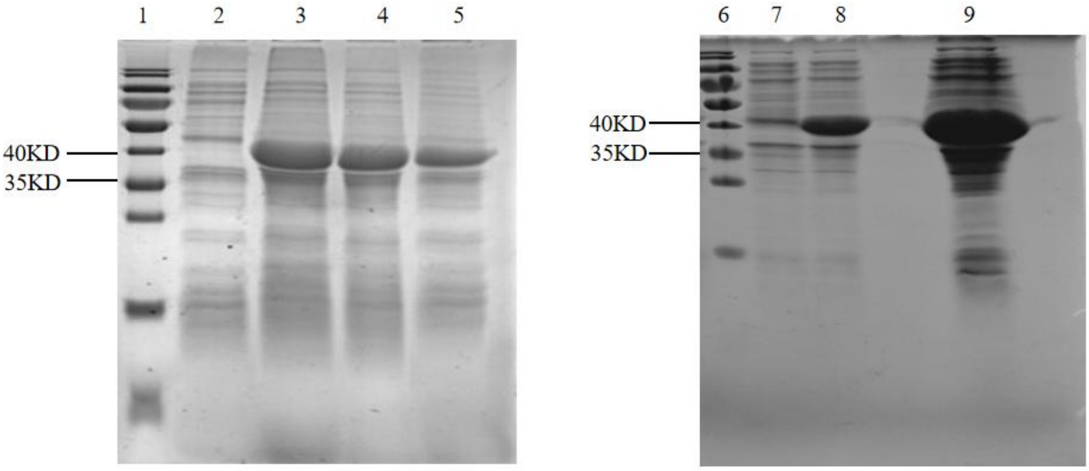

Note：1：Protein standard molecular weight (26617)；2：Uninduced positive bacteria；3：Whole bacteria induced by 0.25 mM IPTG；4：0.25 mM IPTG induced bacterial supernatant；5：0.25 mM IPTG induces bacterial precipitation；6：Protein standard molecular weight；7：Uninduced positive bacteria；8：1 mM IPTG induces bacterial precipitation；9：1 mM IPTG induced bacterial supernatant

Figure 12 SDS-PAGE of pET30a-*cysM* protein expression

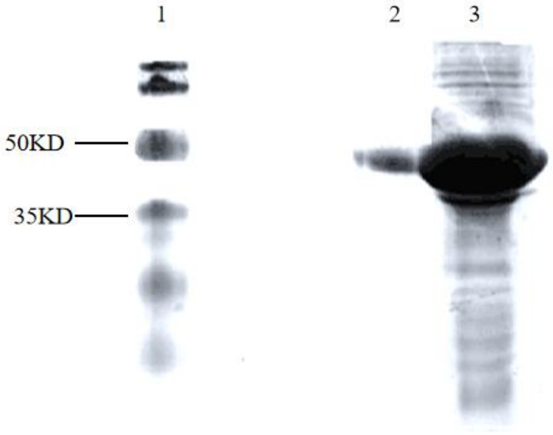

Note：1：Protein standard molecular weight (26617)；2：Purified CysM mature protein；3：1 mM IPTG induced bacterial supernatant

Figure 13 SDS-PAGE of pET30a-*cysM* protein purification
