# Supplementary figures and images for: Tylosin Inhibits Streptococcus suis Biofilm Formation by Interacting With the O-acetylserine (thiol)-lyase B CysM
Source: Front Vet Sci. 2022 Jan 28;8:829899. doi: 10.3389/fvets.2021.829899 (PMC8832016; doi:10.3389/fvets.2021.829899)

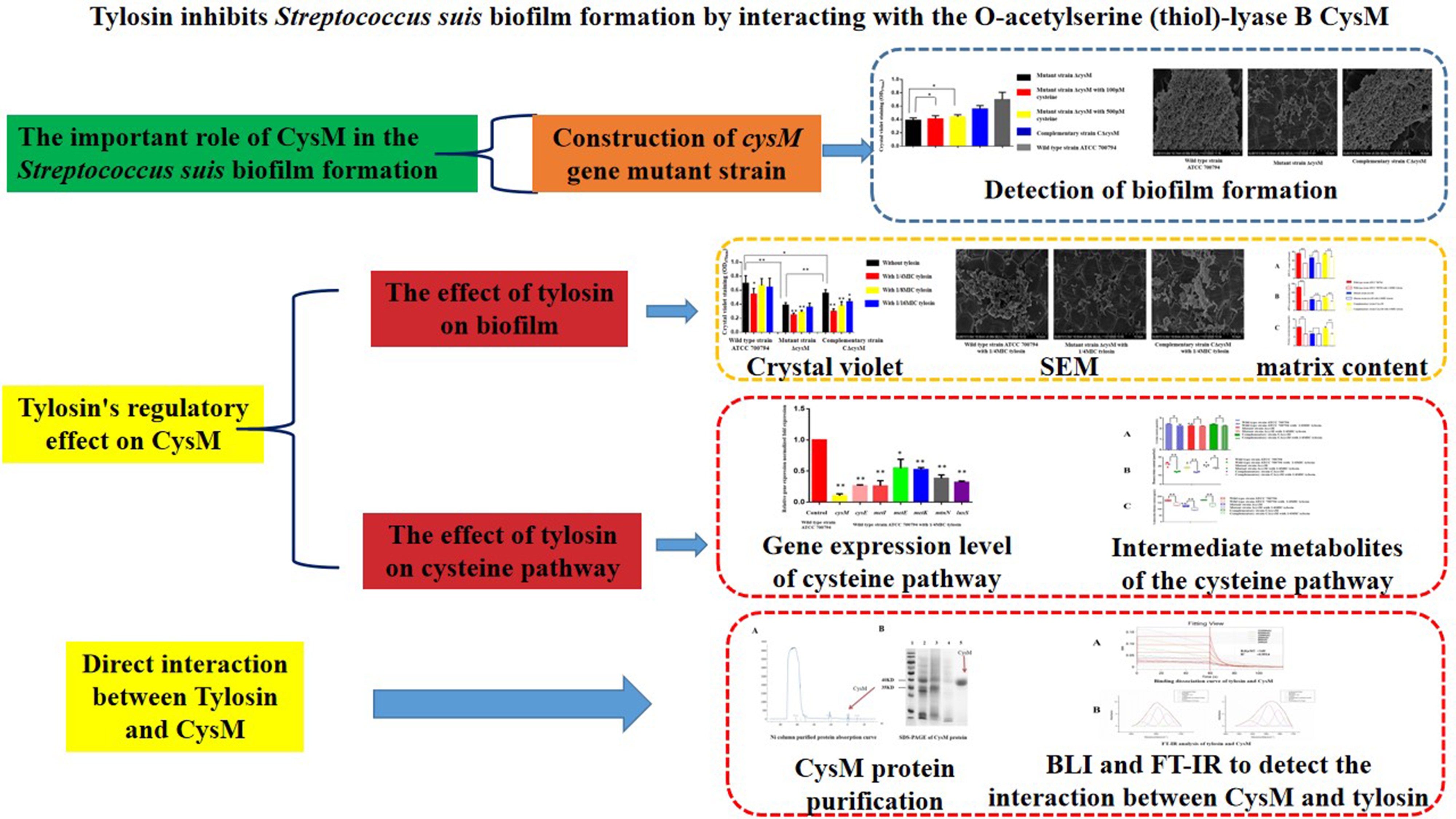

Supplement: Supplementary file 2 [file Image_1.JPEG]

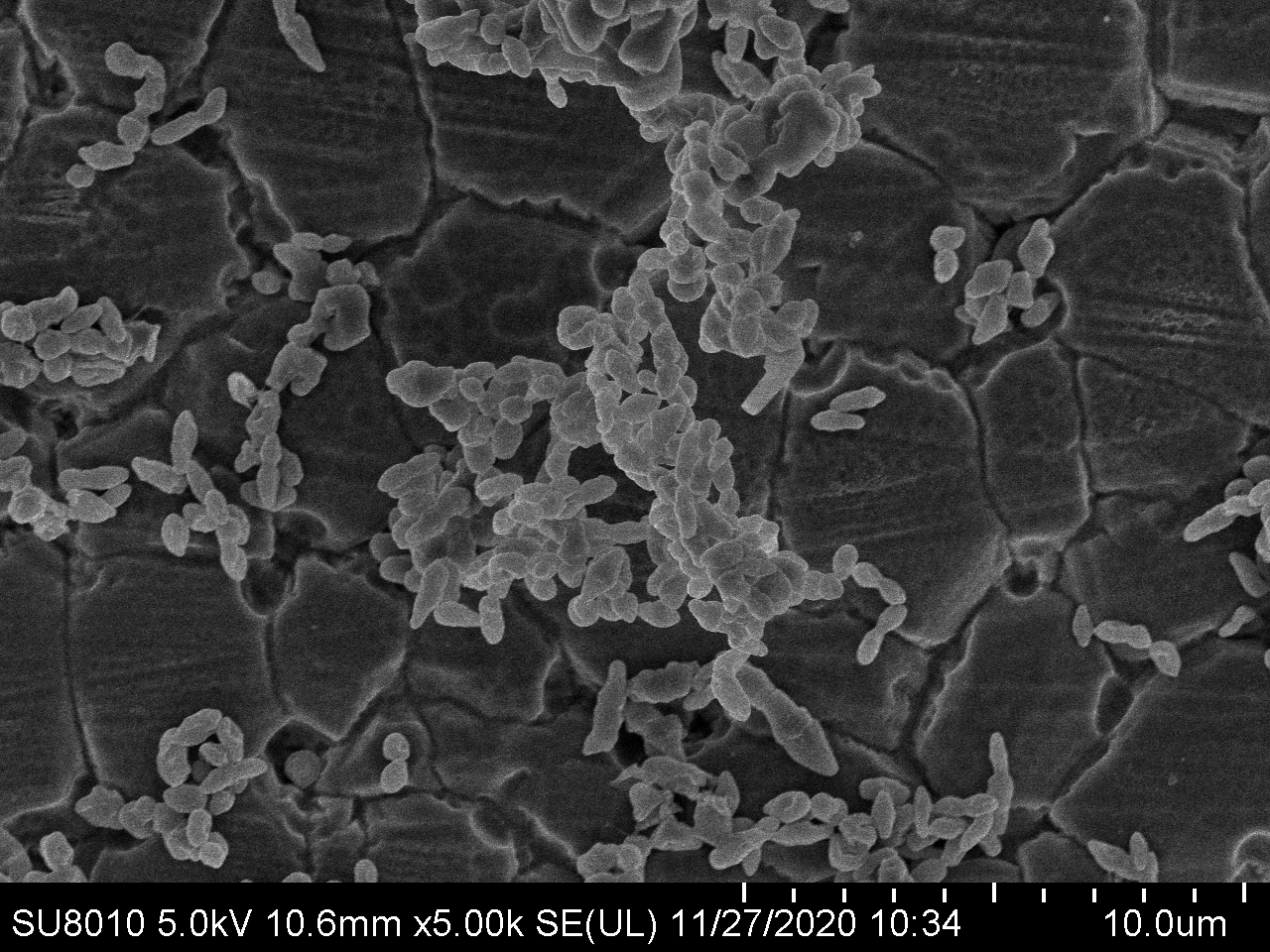

Supplement: Supplementary file 3 [file Data_Sheet_1.zip › Original data/Figure 3 Pictures of scanning electron microscope/CañcysM strain with tylosin.tif]

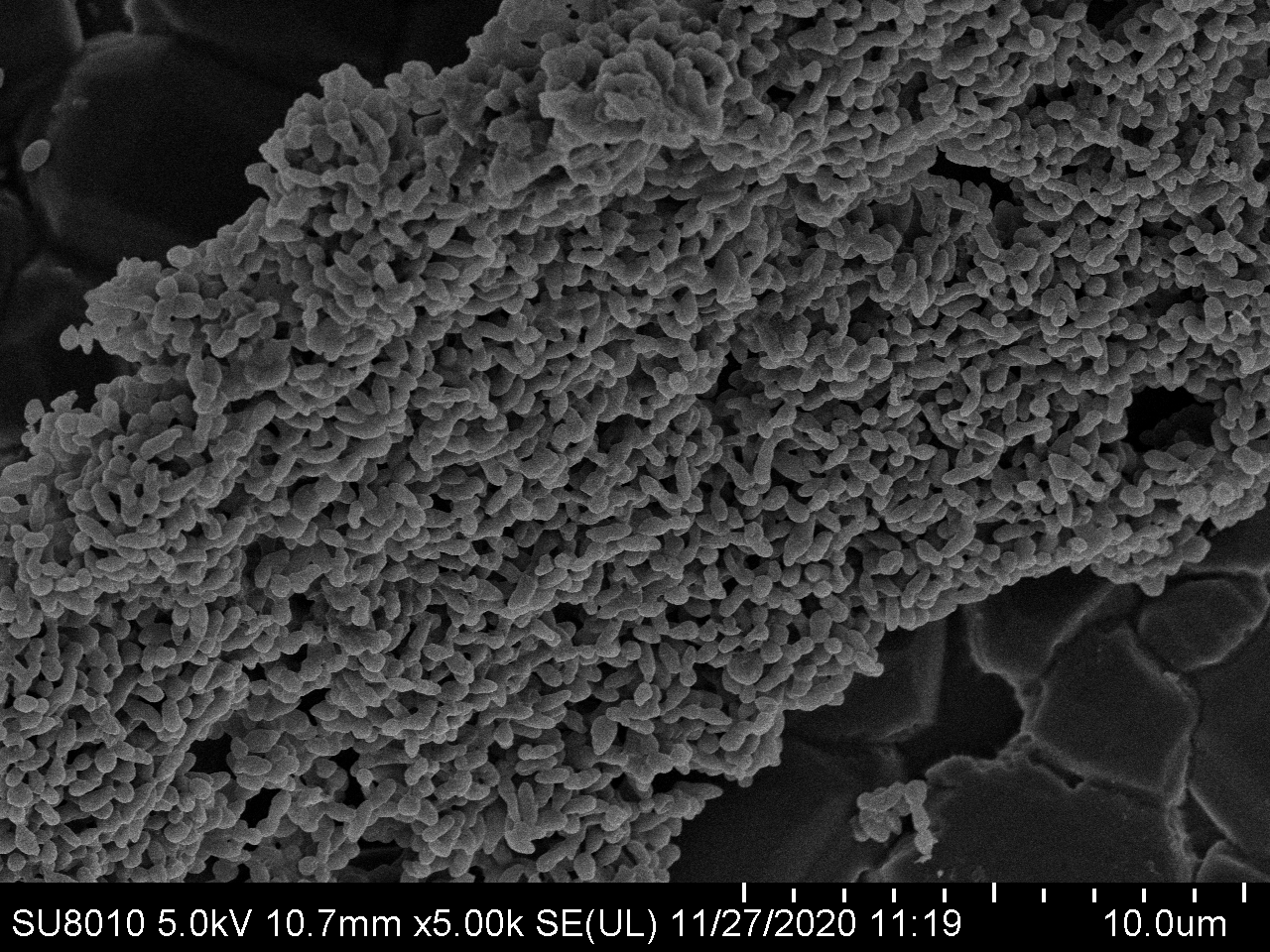

Supplement: Supplementary file 3 [file Data_Sheet_1.zip › Original data/Figure 3 Pictures of scanning electron microscope/CañcysM strain.tif]

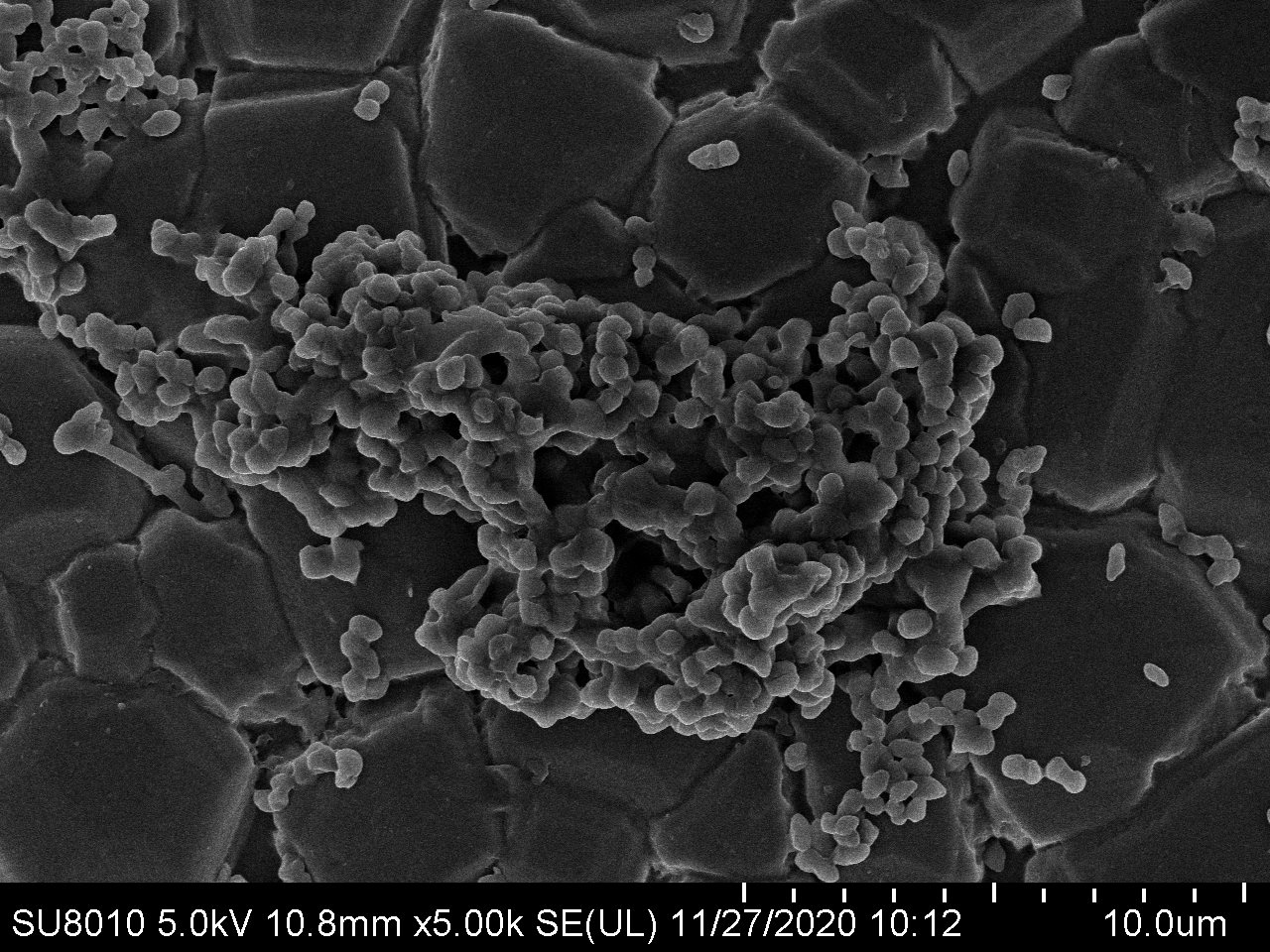

Supplement: Supplementary file 3 [file Data_Sheet_1.zip › Original data/Figure 3 Pictures of scanning electron microscope/WT strain with tylosin.tif]

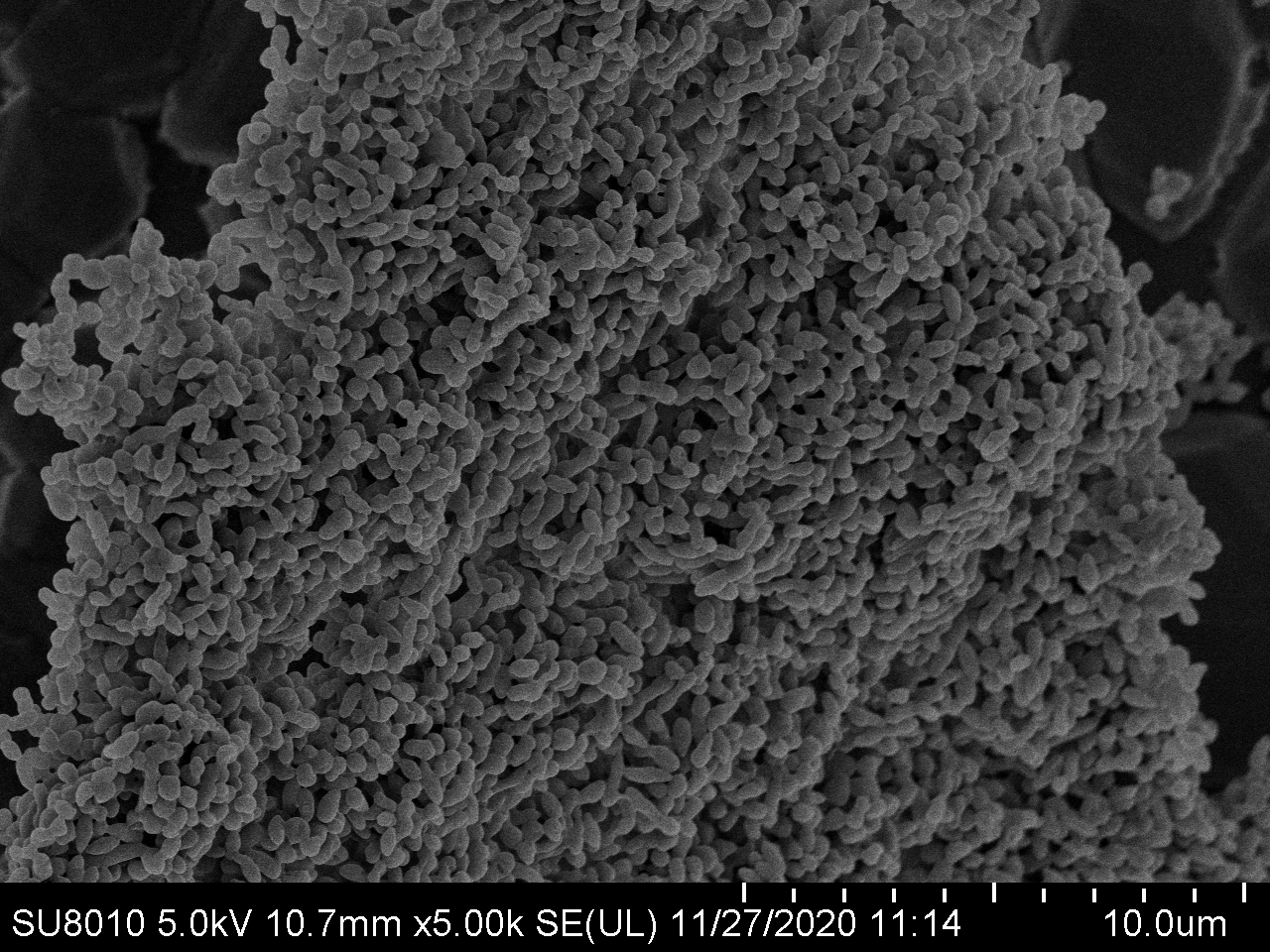

Supplement: Supplementary file 3 [file Data_Sheet_1.zip › Original data/Figure 3 Pictures of scanning electron microscope/WT strain.tif]

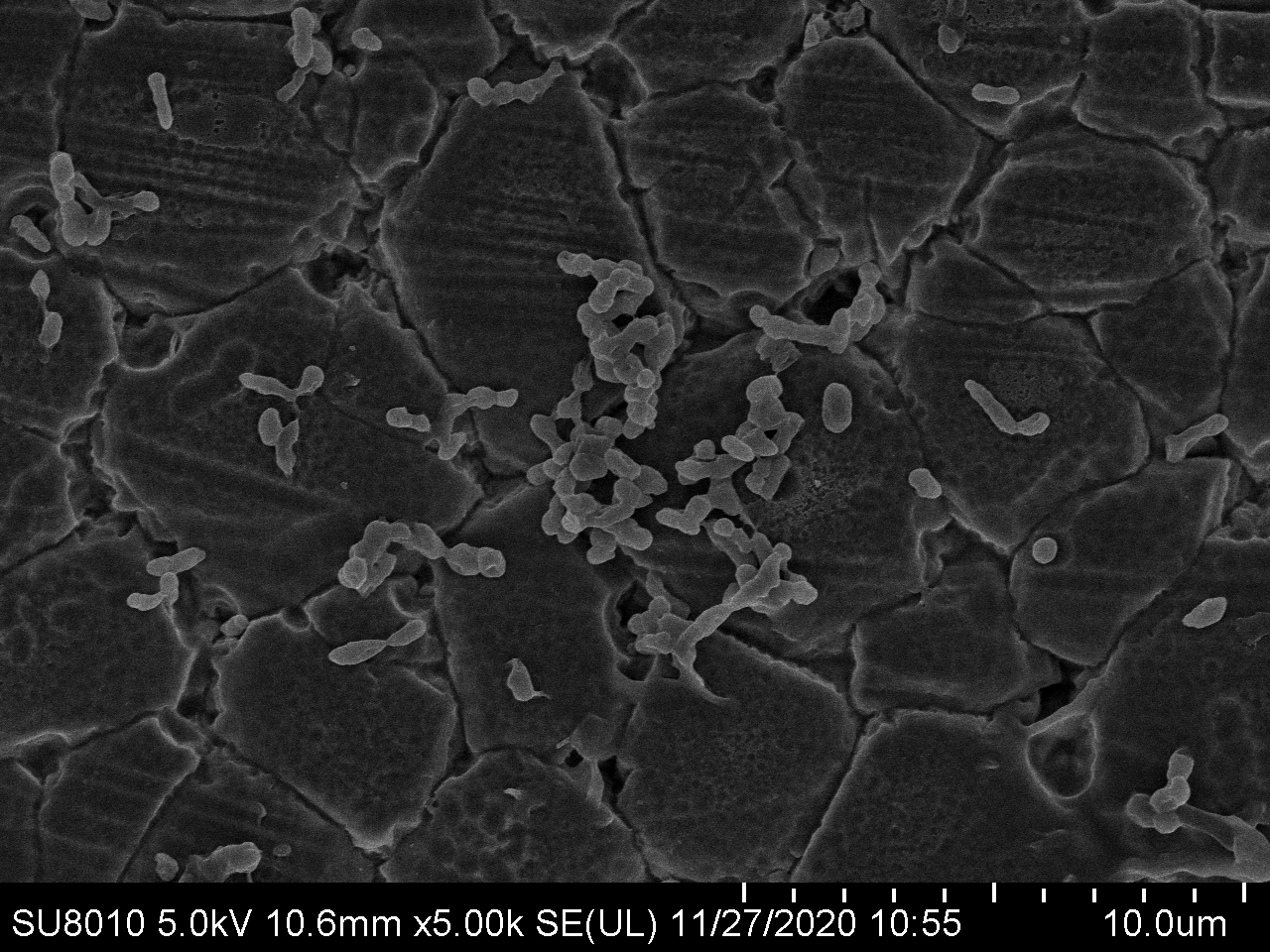

Supplement: Supplementary file 3 [file Data_Sheet_1.zip › Original data/Figure 3 Pictures of scanning electron microscope/añcysM strain with tylosin.tif]

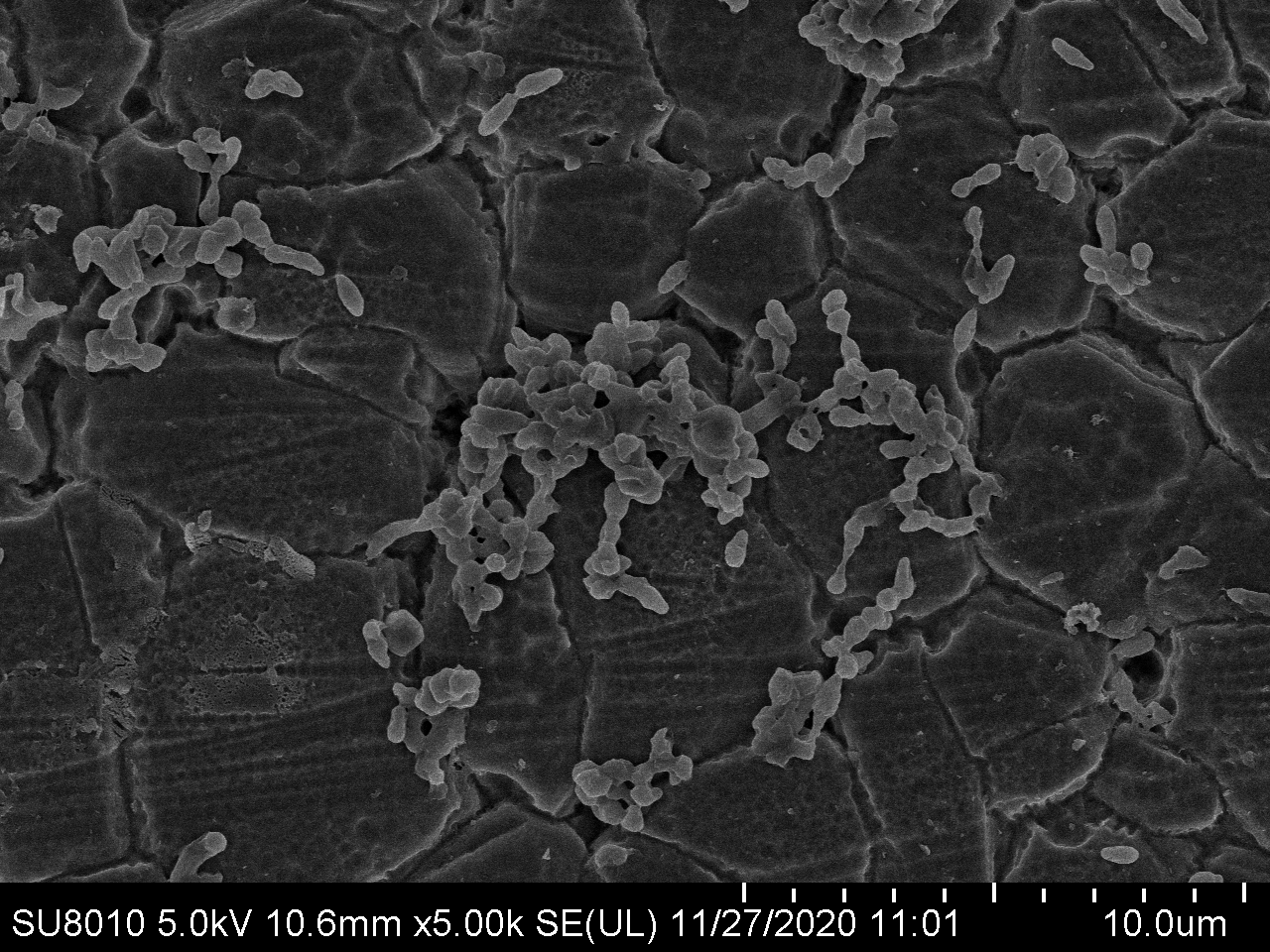

Supplement: Supplementary file 3 [file Data_Sheet_1.zip › Original data/Figure 3 Pictures of scanning electron microscope/añcysM strain.tif]

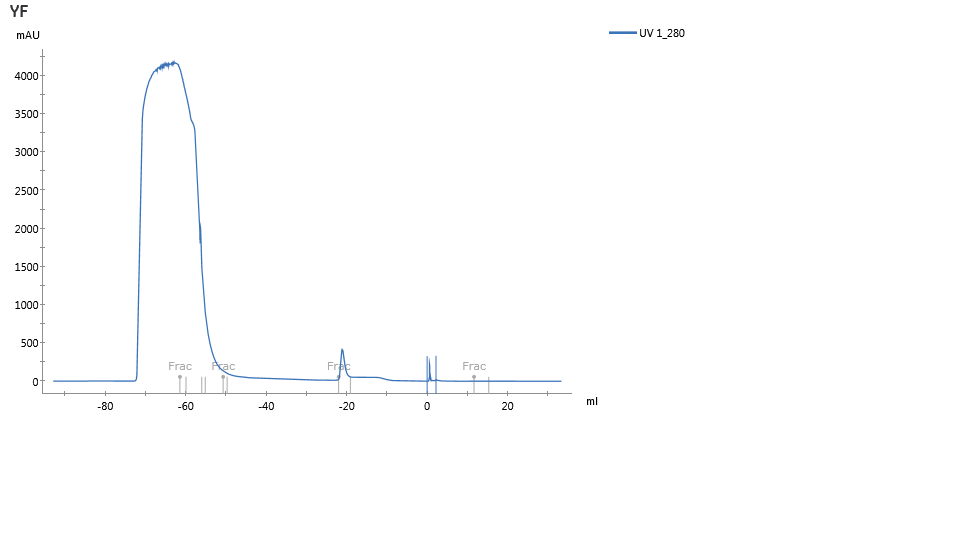

Supplement: Supplementary file 3 [file Data_Sheet_1.zip › Original data/Figure 7 Pictures of protein purification/7A.tif]

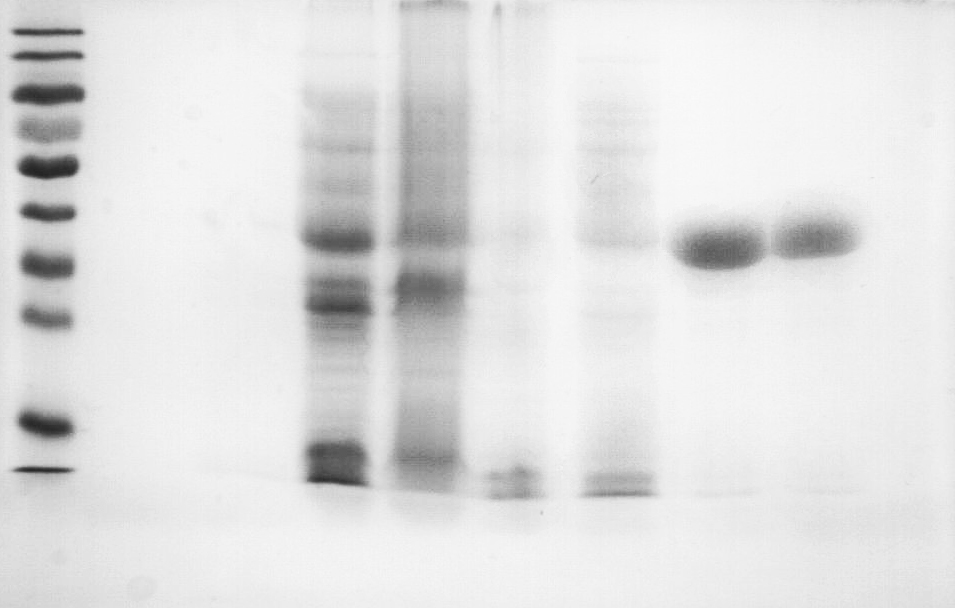

Supplement: Supplementary file 3 [file Data_Sheet_1.zip › Original data/Figure 7 Pictures of protein purification/7B.jpg]

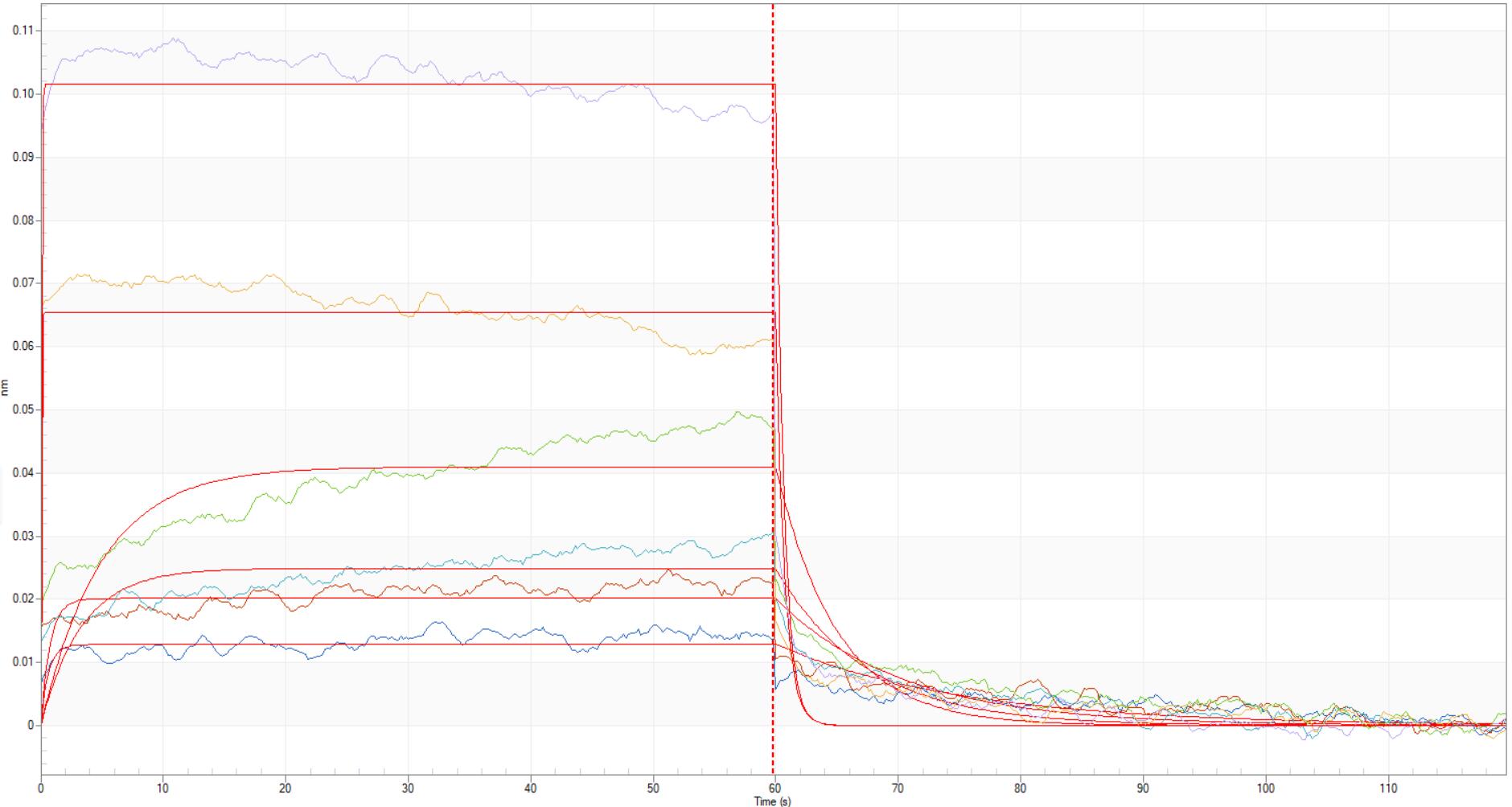

Supplement: Supplementary file 3 [file Data_Sheet_1.zip › Original data/Figure 8A Picture of molecular interaction.jpg]
